# The Intraperitoneal Transcriptome of the Opportunistic Pathogen *Enterococcus faecalis* in Mice

**DOI:** 10.1371/journal.pone.0126143

**Published:** 2015-05-15

**Authors:** Cécile Muller, Margherita Cacaci, Nicolas Sauvageot, Maurizio Sanguinetti, Thomas Rattei, Thomas Eder, Jean-Christophe Giard, Jörn Kalinowski, Torsten Hain, Axel Hartke

**Affiliations:** 1 U2RM-Stress and Virulence, University of Caen Basse-Normandie, EA4655, 14032 Caen, France; 2 Institute of Microbiology, Catholic University of Sacred Heart, 00168, Rome, Italy; 3 CUBE-Division for Computational Systems Biology, Dept. of Microbiology and Ecosystem Science, University of Vienna, 1090 Vienna, Austria; 4 Center for Biotechnology, Bielefeld University, 33594 Bielefeld, Germany; 5 Institute of Medicine Microbiology, Gießen University, 35392 Gießen, Germany; University of Rochester Medical Center, UNITED STATES

## Abstract

*Enterococcus faecalis* is a Gram-positive lactic acid intestinal opportunistic bacterium with virulence potential. For a better understanding of the adapation of this bacterium to the host conditions, we performed a transcriptome analysis of bacteria isolated from an infection site (mouse peritonitis) by RNA-sequencing. We identified a total of 211 genes with significantly higher transcript levels and 157 repressed genes. Our *in vivo* gene expression database reflects well the infection process since genes encoding important virulence factors like cytolysin, gelatinase or aggregation substance as well as stress response proteins, are significantly induced. Genes encoding metabolic activities are the second most abundant *in vivo* induced genes demonstrating that the bacteria are metabolically active and adapt to the special nutrient conditions of the host. α- and β- glucosides seem to be important substrates for *E*. *faecalis* inside the host. Compared to laboratory conditions, the flux through the upper part of glycolysis seems to be reduced and more carbon may enter the pentose phosphate pathway. This may reflect the need of the bacteria under infection conditions to produce more reducing power for biosynthesis. Another important substrate is certainly glycerol since both pathways of glycerol catabolism are strongly induced. Strongly *in vivo* induced genes should be important for the infection process. This assumption has been verified in a virulence test using well characterized mutants affected in glycerol metabolism. This showed indeed that mutants unable to metabolize this sugar alcohol are affected in organ colonisation in a mouse model.

## Introduction

Enterococci are Gram-positive bacteria that colonize several ecological niches, including the gut of mammals and numerous other animals. These bacteria are normally considered harmless or even beneficial for humans, but in the last decades especially two enterococcal species, *Enterococcus faecalis* and *E*. *faecium*, have emerged as important opportunistic pathogens. They are the third and fourth most commonly isolated nosocomial pathogens worldwide, causing up to 14% and 9,6% of hospital acquired infections in the US and Europe, respectively [1–3]. Infections by these bacteria are difficult to treat due to intrinsic and acquired resistances to major classes of antibiotics. Enterococci (especially *E*. *faecalis*) are considered as “hard” bacteria due to their capacity to survive in harsh environments which are lethal for other bacteria [4,5] including the survival in a dessicated state on surfaces for months [6]. Non-growing cells of *E*. *faecalis* develop multiple, non-specific stress resistances that increase their survival capacity to lethal conditions by several orders of magnitude compared to growing cultures [7]. Altogether, the remarkable intrinsic ruggedness and resistance to antibiotics of enterococci is supposed to be the reason why they so successfully persist and spread within health care settings.

In the last 20 years, most work has been done with *E*. *faecalis* to understand the basis of virulence and to identify targets for new treatment strategies such as vaccination. This is rather challenging since *E*. *faecalis* is not so inherently virulent like other pathogens. Nevertheless, in several well controlled animal models a dozen of potential factors have been proposed to be important for virulence or at least to contribute to it [8]. Major virulence factors of these candidates correspond to (i) extracellular matrix proteins like aggregation substance (AS), enterococcal surface protein (Esp), the *E*. *faecalis* antigene (EfaA), the adhesion to collagen (Ace), the endocarditis and biofilm associated pili (EbpA pili) and (ii) to cell and tissue damaging activities like the bacteriocin cytolysin (Cyl) and the protease gelatinase (GelE) [8]. However, neither of these traits could be systematically identified in clinical isolates.

Genome sequencing of clinical and commensal isolates have accelerated the identification of potential virulence traits. This led to the discovery of a pathogenicity island (PAI) of greater than 150 kbp in *E*. *faecalis* including genes encoding the known virulence factors Esp, cytolysin and aggregation substance [9]. Other genes encode putative adhesins and invasins, exoenzymes, proteases and surface and extracellular proteins. However, virtually nothing is known about the implication of these new traits in the infection process [10].

A crucial but hitherto neglected aspect of the infection process of enterococci concerns also proliferation after infection. These polyauxotophic bacteria need complex media for *in vitro* growth and they have to find or to actively mobilize all essential growth factors once inside the host. Furthermore, nothing is known about the energy sources and the catabolic pathways used by enterococci during infection. Collectively, this demonstrates that our knowledge about the real world infection process of enterococci is still limited. In order to get a deeper insight into enterococcal virulence, whole genome expression profiles of cells isolated from infection sites and comparison of the results with the transcriptional activities of *in vitro* cultured cells are needed. This should allow the identification of genes actually differentially expressed *in vivo*. The recently introduced massively parallel cDNA sequencing (RNA-seq) techniques becomes now the gold standards for the sensitive determination and accurate quantification of transcriptomes [11–13] although hitherto only few studies analysing gene expression of a pathogen during infection of mammalian hosts have been published [14–19]. In this work, we will present the first comprehensive *in vivo* expression database of a clinical isolate of *E*. *faecalis* obtained by RNA-seq.

## Materials and Methods

### Bacterial strains and growth media

*E*. *faecalis* V19 strain (plasmid-cured V583 strain) or JH2-2 were used as wild-type strains (WT). *E*. *faecalis* bacteria were grown at 37°C in brain heart infusion (BHI) to mid-log phase (OD_600nm_ = 0.6) or stationary growth phase after 24h culture.

### Bacterial infection of mice

For *in vivo* RNA preparation, enterococcal cells were grown as standing cultures at 37°C in BHI and then pelleted and resuspended in 5 ml of phosphate-buffered saline (PBS) for injection. BALB/c mice were infected by intraperitoneal injection of 500 μl of the suspension of 10^9^ to 10^10^ cells. After 24 h of infection, the peritoneal macrophages and bacteria were collected by two peritoneal washes, each with 5 ml of PBS. The cell suspension was then lysed with detergent, washed three times with PBS and used for RNA extraction.

The virulence of JH2-2 (wild-type strain) and the deletion mutants was also assessed in an intravenous infection model. Briefly, overnight cultures of the strains grown in BHI supplemented with 40% heat-inactivated horse serum were centrifuged, and the resulting pellets were resuspended in sterile PBS to achieve final concentrations of 10^9^ bacteria/ml. Aliquots of 100 μl from each strain were used to inject each of 10 female BALB/c mice (10 weeks old; Harlan Italy S.r.l., Udine, Italy) through the tail vein. Infection experiments were repeated three times. Mice were monitored twice daily and were euthanized by CO_2_ asphyxiation 7 days after infection. Kidneys and livers were then removed aseptically, weighed, and homogenized in 5 ml of PBS using a stomacher (model 80; Pbi International, Milan, Italy) for 120 sec at high speed. For CFU determination, serial homogenate dilutions were plated onto Enterococcus selective agar (Fluka Analytical, Switzerland). CFU counts were analyzed by the unpaired *t*-test.

### Ethic Statement

The mouse experiments were performed under a protocol approved by the Institutional Animal Use and Care Committee at the Università Cattolica del Sacro Cuore, Rome, Italy (Permit number: Z21, 11/01/2010) and authorized by the Italian Ministry of Health, according to the Legislative Decree 116/92, which implemented the European Directive 86/609/EEC on laboratory animal protection in Italy. Animal welfare was routinely checked by veterinarians of the Service for Animal Welfare.

### RNA purification

RNA was harvested from bacteria grown in BHI medium to mid-exponentially (OD_600nm_ = 0.6) or stationary growth phase (24 h of culture) and from bacteria grown inside mice peritoneum. Pellets were resuspended in 200 μl of Max Bacterial Enhancement Reagent (Invitrogen). This suspension was added to a 2:1 volume of acid phenol (Ambion) with glass beads, and then agitated during 30 min at 30 Hz in the shaker MM200 (Retsch GmbH, Haan, Germany). After centrifugation, aqueous phase is transferred in 1 volume of TRIZol (Invitrogen), mixed and incubated 5 min at room temperature. One volume of ethanol is then added. RNA was then purified using Direct-Zol RNA Miniprep (Zymo Research, Irvine, USA CA) according to the manufacturers’ instructions.

### cDNA synthesis

Genomic DNA removal and cDNA synthesis were assessed by using the Quantitect Reverse Transcriptase kit (Qiagen) on 3 μg of RNA, according to the manufacturers’ instructions.

### RNA-Seq and reads mapping

From the total RNA samples, the small RNA species < 500 nucleotides were isolated using the mirVana miRNA isolation kit (Ambion). The isolated RNA fraction was first treated with Terminator Exonuclease (Epicentre) as recommended by the manufacturer in order to enrich for primary transcripts. Then the small RNA were poly(A)-tailed using poly(A) polymerase and 5’-triphosphate of RNA was removed with tobacco acid pyrophosphatase (Epicentre) as recommended by the manufacturer. This treatment allows discriminating primary 5’-ends generated by transcription initiation from 5’-ends generated by RNA processing. Afterwards, an adapter (5’-AATGATACGGCGACCACCGACAGGTCAGAGTTCTACAGTCCGACGATC-NNNN) was ligated to the 5’-monophosphate of the RNA. First-strand cDNA synthesis was performed using an oligo(dT)-adapter primer (5’-CAAGCAGAAGACGGCATACGATTTTTTTTTTTTTTTTTTTTTTT) and M-MLV reverse transcriptase (Promega) assay. The resulting cDNA were PCR-amplified in 12–15 cycles to a concentration of 20–30 ng/μl using a high fidelity DNA polymerase. The 5’-barcoded cDNA were purified using Agencourt AMPure XP kit (Beckmann Coulter Genomics). Ready-to-sequence cDNA libraries were constructed by Vertis Biotechnologie AG (Freising, Germany). Samples were pooled at approximately equimolar amounts (2 pmol each) and sequencing was performed on an Illumina GAIIx sequencer (Illumina, Inc., San Diego, CA) producing single reads of 75 bases in length.

The raw sequencing reads were de-multiplexed using fastx_barcode_splitter [20] and the bar-codes were removed using fastx_trimmer[20]. The poly-A tails were removed with cutadapt [21] (parameters:-e 0.1-b AAAAAAAAAAAAAAAAAAAAAAAAA-b TTTTTTTTTTTTTTTTTTTTTTTTT-f fastq) and trimest [22] using default parameters. Quality trimming and length filtering was done via fastq_quality_trimmer (parameters:-Q33-v-l 20-t 20) followed by quality filtering with fastq_quality_filter [20] (parameters:-Q33-v-q 20-p 85). The remaining high quality sequence reads were mapped to the *Enterococcus faecalis* reference genome (GenBank accession no. AE016830) and to the human and mouse genomes (GenBank accession no. GCA_000001405.11 and GCA_000001635.3) using BWA [23]. The post-processing was done using Samtools [24]. The resulting sorted BAM files were the input for the peak detection via MACS [25] (parameters:-g 3.22e+06—shiftsize = 36—nomodel). The coverage of the detected peaks was calculated using a custom perl script (available on request) resulting in a list of coverage per peak. The normalization via TMM [26] and the following differential expression analysis was carried out using NOISeq [27].

The positions classified as differentially expressed were the input for manual investigation via IGV [28,29]. Only genes with homogeneous coverage were considered for the following steps. The maximal count value of reads overlapping each gene base was recorded. To remove bias due to background signal, we chose to consider CDS with reads count superior to 200.

### RNA-Seq data accession number

RNA-Seq data from this study are available in NCBI SRA repository, reference number PRJNA276169.

### Quantitative PCR (qPCR)

Primers were designed using Primer3 (http://frodo.wi.mit.edu/primer3/). Forward (F) and reverse (R) primers used for qPCR are listed in S5 Table. PCR reactions were conducted with the GoTaq DNA polymerase (Promega), according to the manufacturer’s instructions. Triplicate qPCR reaction were performed on 10 ng of cDNA per reaction, with 1 μM of primers and the GoTaq qPCR Master Mix (Promega), according to the manufacturers’ instructions. Reactions were loaded into a CFX96 Real-Time PCR detection system (BioRad) and the PCR were performed with the following conditions: 95°C for 3 min, followed by 40 cycles at 95°C for 15sec, 60°C for 1 min. The transcription level of each gene was normalized to that of the rRNA 5S reference gene. Control genomic DNA from WT cells was used to construct standard curves.

### RT-PCR experiments

For reverse transcriptase PCR (RT-PCR) experiments, two micrograms of RNA was reverse transcribed with random hexamer primers and QuantiTect enzyme (Qiagen) according to the manufacturer’s recommendations. cDNAs were then used as templates for PCRs using primers listed in S2 Table. PCR were performed with the GoTaq DNA polymerase (Promega) and with the following conditions: 95°C for 3 min, followed by 30 cycles at 95°C for 30sec, 56 to 60°C for 30 sec and 72°C for 30 sec. PCR products were separated on agarose gel.

## Results and Discussion

### Identification of genes induced under infection conditions by RNA sequencing

In order to identify genes induced during *in vivo* infection using RNA-Seq, RNA was prepared from *E*. *faecalis* V19 grown to mid-log phase or stationary phase in BHI broth and from cells incubated 24 h in the mice peritoneum and sequenced on the Illumina GAIIx platform.

In total, we analysed 14,219,699 sequences (total number of reads). Reads size were between 70 and 100 bp for all conditions, and we mapped 3,102,984; 4,111,728 and 5,136,137 reads to *Enterococcus faecalis*, for laboratory culture (exponential and stationary growth phases) and *in vivo* conditions, respectively. In all datasets only few reads were mapped to the human and mouse genomes: 972 (0.03%), 4,101 (0.10%) and 587 (0.01%), respectively.

**Fig 1:**
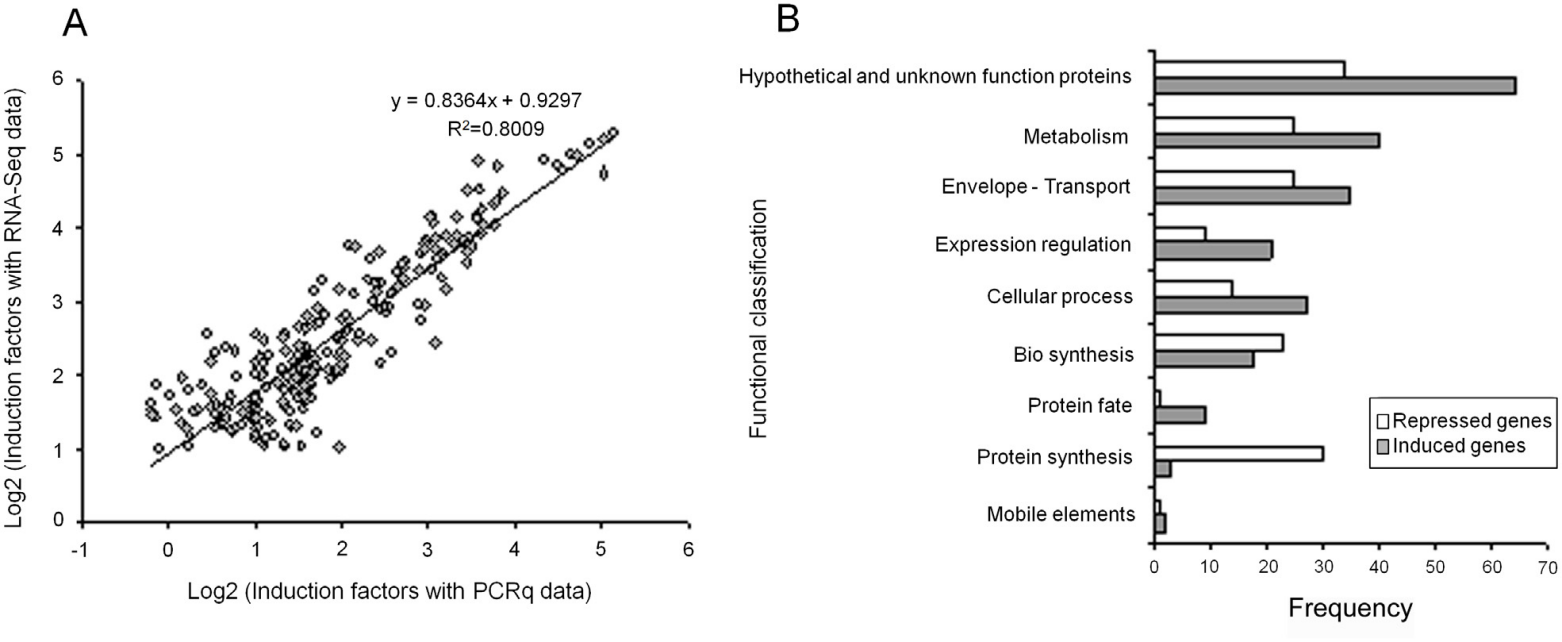
Overview of *in vivo E*. *faecalis* transcriptome analyzed data. (A) Correlation between induction factors of *in vivo* expressed genes obtained with RNA-Seq data and qPCR data, comparing genes expressed during peritonitis and mid-log phase culture in BHI. (B) Bar graph displaying differentially expressed genes of *E*. *faecalis* during mouse peritoneum infection according to functional categories. White and grey bars indicate genes with repressed and induced expression *in vivo* compared to cells grown to mid-log phase in BHI, respectively.

We used these data as a first screen for the identification of differences in gene expression with a special emphasis to identify genes induced during mice peritonitis. For comparative quantification of gene expression, read counts were determined for every CDS. CDS with at least 200 mapped reads were considered for expression analysis. The threshold for a classification as differently expressed was 2-fold. CDS with *p*<0.05 were considered significantly deregulated. Moreover, the data allowed also the mapping of 192 transcriptional start sites (S1 Table). Since the physiological state of the bacteria inside the mouse peritoneum is unknown, we compared the *in vivo* gene expression to the RNA-Seq data of exponentially growing and of stationary phase cells. This showed that 273 and 223 CDS seemed to be significantly induced and 170 and 154 CDS were repressed *in vivo*, respectively (S1 Table). The *in vivo* induced CDS were then checked by RT-qPCR using 3 independent RNA extractions from cells isolated from the mouse peritoneum and mid-log phase cultures as the comparator to confirm the validity of RNA-Seq data. This showed that 77% of the initial CDS were confirmed (*p*-value<0.05). Induction factors from RNA-Seq data and RT-qPCR were well correlated (R^2^ = 0.80) (Fig 1A), supporting that RNA-Seq provided reliable quantitative estimates of expression for CDS with read counts ≥200 and induction factor >2. The genomic environment of each CDS was analyzed, and putative operon structures were determined and checked by RT-PCR experiments (S1 Fig, S2 Table).

The combined results were used to establish tables of candidate genes that might have *in vivo* induced or repressed expression (S3 and S4 Tables, respectively) when compared to both expressions in exponential growing or stationary phase cells. These genes were then classified into functional categories based on NCBI cellular roles (http://www.ncbi.nlm.nih.gov) (Fig 1B, Table 1). The functional profiles of these sets differed greatly between peritoneal infection or laboratory culture conditions. Thus, 211 CDS expressions were significantly induced *in vivo*, and 157 were repressed. It should to be noted that 68% of the induced and 85% of the repressed genes showed a change in their expression by a factor greater than 5-fold.

**Table 1:** Functional classification of induced and repressed genes during mice peritoneum infection.

Cellular role categories	Functional subcategories	Induced genes	Repressed genes
**Envelope and transport** *including*:		**34**	**25**
	ABC transporters	17	9
	PTS systems	1	6
**Metabolism** *including*:		**40**	**24**
	Gycolysis	2	2
	Fermentation	12	3
	DNA, RNA and nucleotides	8	11
	Amino acids and polysaccharides	6	0
**Biosynthesis** *including*:		**17**	**23**
	Amino acids and polysaccharides	6	2
	Wall	1	18
**Proteins synthesis** *including*:		**3**	**28**
	Ribosomal proteins	2	24
**Cellular process**		**27**	**14**
	Stress response	13	2
	Virulence	11	1
	Division	0	9
**Protein fate**		**8**	**1**
**Expression regulation** *including*:		**21**	**9**
	Transcription	13	8
	Signal transduction	7	0
**Mobile elements**		**2**	**1**
**Hypothetical proteins**		**45**	**29**
**Unknown function**		**14**	**3**
**Total**		**211**	**157**

The largest group of *in vivo* induced genes (59 genes) encodes hypothetical or unknown function proteins with induction factors between 2 and >30 fold. This illustrates how little we seemingly know regarding the opportunistic mechanisms of *E*. *faecalis*. The other functional classes that are highly represented are “Metabolism” (40 genes), “Cellular process” (27 genes), and “Envelope” (34 genes). Genes encoding proteins with regulatory functions represents 21 genes induced suggesting that an important reorganization of gene expression occurs during *in vivo* infection. Concerning repressed genes, the most striking decrease of expression is observed for genes encoding ribosomal proteins (24 genes) and enzymes involved in biosynthesis of cell wall components (18 genes) or cell division protein (9 genes). These data suggest that *E*. *faecalis* cells are not actively dividing during peritonitis. We verified this further by monitoring bacterial persistence in the mouse peritoneum periodically over 24 hours. This showed that CFU counts reisolated from the peritoneum do not change significantly over time (S2 Fig).

### Induction of genes involved in virulence during mice peritonitis

If the above described analysis reflects the livestyle of *E*. *faecalis* under infection conditions, previously described genes encoding major virulence and virulence-associated factors should be part of the *in vivo* induced genes.

The cytolysin (or haemolysin) is a major secreted virulence factor of *E*. *faecalis* [30]. Expression of structural genes encoding the cytolysin subunits (*cylL*_*L*_—*ef0525* and *cylL*_*S*_—*ef0526*) and post-translational modification protein (*cylM*—*ef0527*) is induced +7.4-fold in our model of infection. The expression of the cytolysin has been associated with increased toxicity in different models of infections, including human bacteremia or mouse intraperitoneal infection [31]. In the *E*. *faecalis* V19 strain as well in its parent strain V583, the cytolysin operon is located on the PAI of the *E*. *faecalis* strain V583 [31] but lacks *cylA* and *cylI* genes and the *cylB* gene is truncated. However the two-component regulatory system *cylR1-cylR2* is present, which means that an autoinducer-independent regulation of the operon expression is functional, although cytolysin is not produced.

Several major components of the bacterial cell envelope contribute to virulence. For example, the endocarditis-specific antigen EfaA (EF2076) [32] is the third gene of an operon (*efaCBA*) encoding an ABC transporter. Since EfaA is up-regulated under Mn^2+^ limiting conditions, it has been proposed that the *efaCBA* operon encodes a Mn^2+^ transporter, with EfaA being the putative substrate-binding lipoprotein component [33]. Using a mouse peritonitis model, Singh *et al*. [34] showed that infection with an *efaA*-negative mutant led to prolonged survival of mice compared to infection with a wild-type *E*. *faecalis* strain. Our data indicate that the expression of this operon is induced +6.9-fold in the peritonitis infection model.

The CDS *ef1818* encoding the extracellular metalloendopeptidase coccolysin or gelatinase (GelE) is located immediately upstream of the *sprE* gene encoding a serine protease. The *ef1818-ef1817* operon and especially the gene encoding gelatinase contributes to the severity of infection in *E*. *faecalis* [34,35], and our results show that it is induced +7.7-fold *in vivo*. Expression of this locus is regulated by the quorum-sensing two-component system Fsr, encoded by the operons *fsrA* (*ef1822*) and *fsrBCD* (*ef1821-ef1820)* [36]. Each *fsr* transcription unit is induced *in vivo* 3.5- and 3.3-fold, respectively. The *fsrABC* system seems also to be involved in the modulation of abundance of the virulence relevant adhesin Ace on the cell surface (EF1099) [37]. Ace is a MSCRAMM (Microbial Surface Components Recognizing Adhesive Matrix Molecules) protein and was shown to bind to collagen [38]. Modulation by FsrABC of surface exposed Ace is likely due to expression control of gelatinase by the *fsr* operon [37]. This same study demonstrated that Ace is cleaved from the cell surface by this protease. Of note in this context is that Ace is significantly induced (+4.6-fold) in our peritonitis model supporting also a previous finding that Ace is actively expressed in cells isolated from infective endocarditis vegetations [38].

Finally, the aggregation substances (AS), a highly conserved family of surface-anchored polypeptides, have adherence properties [39] that enable *E*. *faecalis* to interact with host cells and extracellular matrice proteins. Furthermore, AS is associated with increased virulence and mortality in infective endocarditis [40] or peritonitis models [41]. The *E*. *faecalis* V583 strain harbors two genes encoding this type of adhesine, one in the core genome (*ef0149*) and one in the pathogenicity island (*ef0485*). Gene *ef0149* is significantly overexpressed in our infection model (+5.3-fold), while there is no modification of expression detected for *ef0485*.

We concluded from the combined results that the *in vivo* expression database reflects well an infection process.

### *E*. *faecalis* is exposed to stresses during peritoneum infection

Phagocytic cells infiltrate the peritoneum several hours after infection [42]. After phagocytosis, microorganisms are subjected to diverse stresses inside the phagolysosomes. Therefore, operons encoding stress proteins should be among the selected genes induced during peritonitis.

Our data demonstrate that this is indeed the case. Several genes encoding known or suspected general stress proteins [EF0453 (Ohr or Gsp65 [43]), EF0770 (Gsp62 [44]), and EF1058 (Universal Stress Protein) and EF3233 (Dps [45])] are overexpressed +3.4-, +4.9-, +90.8-, and +2.6-fold *in vivo*, respectively. Also genes encoding class 1 (molecular chaperons) and class 2 (proteases) heat shock (HS) proteins are strongly induced. In the case of the class 1 HS genes encoding chaperon systems, the *hrcA-grpE-dnaK-dnaJ* (*ef1306-ef1310*), *groEL-groES* (*ef2633-ef2634*) and *tig* (*ef0715* encoding trigger factor [46]) operons have an induced expression of +13.6-, +7.2- and +3.8-fold, respectively. Further evidence that cells seem to cope with protein folding stress during their presence in the peritoneum is illustrated by the induction of the protease ClpP (EF0771) overexpressed +3.4-fold and of ClpX (EF1917) one of its possible ATPase subunits which is induced +6.3-fold. In addition, the gene encoding the ClpP-independent disaggregase *clpB* (*ef2355*) [47] has an induced expression of +3.9-fold *in vivo*.

The main bactericidal mechanism of phagocytic cells is based on the intentional synthesis of reactive oxygen species (ROS) inside the phagolysosomes with the aim to kill invading microorganisms. *E*. *faecalis* survive inside peritoneal macrophages by several orders of magnitude better than *E*. *coli* [48]. This demonstrates that it can cope well with exposure to ROS. It harbors three different NADH-dependent peroxidases, the NADH peroxidase Npr (EF1211), the alkyl hydroperoxide reductase AhpCF (EF2739-EF2738), and the thiol peroxidase Tpx (EF2932). The cellular roles of these activities have been previously defined [49] and this demonstrated that the most important activity for the survival inside peritoneal macrophages is Tpx. The corresponding mutant was also tested in a mouse peritonitis model and this showed that it was the most attenuated strain in comparison to the wild-type, the *ahpCF* and *npr* mutant strains. Beside the peroxidases, *E*. *faecalis* possesses other main antioxidative activities such as a manganese-cofactored superoxide dismutase encoded by the *sodA* gene (*ef0463*) which has been shown to be important for the survival inside macrophages [50] and a monofunctional heme-dependent catalase encoded by the *katA* gene (*ef1597*) [51].

We screened our *in vivo* induced genes database for the presence of the genes encoding these antioxidative activities and noted that it reflects well the former findings. Indeed, the most *in vivo* induced peroxidase is by far Tpx. Expression of the gene encoding this enzyme is induced 14.8-fold whereas Npr and AhpCF are more moderately induced (+3.7- and +3.2-fold, respectively). The expression of *sodA* is also somewhat induced *in vivo* (+3.2-fold). Moreover, several other genes with known or suspected implications in oxidative stress are present in the *in vivo* induced gene database such as genes endoding thioredoxin reductase (*ef1405*; +4.8-fold), two members of the ferric uptake regulator family, PerR (*ef1585*; 4.4-fold) [52] and Fur (*ef1525*; +5.9-fold), and the Dps family protein (*ef3233*; +2.6-fold) [45] as well as the *arc* operon implicated in arginine metabolism (*ef0104-ef0108*, +7.9-fold) [53] and the *sufCDSUB* operon involved in the assembly of Fe-S clusters (*ef2390-ef2394*; +7.4 fold)[54–56].

### Gene expression in mobile elements during peritonitis

*E*. *faecalis* has exceptional adaptation capacities to the clinical environment by acquisition of antibiotic resistance and pathogenicity traits. A large proportion of the genome of the V583 strain corresponds to mobile genetic elements including 7 phages [57] and a PAI (genes *ef0479* to *ef0628* in the V583 sequence [9]). In general, few PAI genes are differently expressed in our infection model. As mentioned before, the enterococcal cytolysin is significantly induced *in vivo* (*ef0525-ef0528;* +7.4-fold) (S3 Table) but the most induced operon of the PAI encodes a putative cation ABC transporter (*ef0575-ef0578*; +22.1-fold). Since the last gene of this operon is annotated as an iron-dependent repressor, the system may be implicated in iron transport. Moreover, 3 genes encoding TetR family transcriptional regulators (*ef0579* and *ef0600-ef0601*) are significantly induced (+4.6- and +6.9-fold, respectively).

The V19 genome harbors 7 prophages (pp1 to pp7) and their dynamics and contributions to pathogenic traits have been recently studied [57]. pp1 is enriched in clonal complex 2 isolates, which are particularly well adapted to hospital environment and associated with invasive disease [58]. The capacity of *E*. *faecalis* V19 to bind to human platelets seems to be particularly linked to the presence of pp1 [57]. Our data indicate that the expression of 5 genes of pp1 (*ef0332* to *ef0336)* are induced during peritonitis (+13.2-fold). However, the protein which likely mediates *E*. *faecalis* binding to platelets (*ef0348*) was not part of these induced genes [57].

### Metabolic activities of *E*. *faecalis* during peritoneum infection

After infection, bacteria have to proliferate inside the infected host. This is particulary challenging for polyauxotrophic microorganisms like enterococci since these organisms need a large panel of nutrients for growth. Either the bacteria get all these growth factors from the host or they have mechanisms to mobilize them actively. An important question concerns also the energy source used by enterococci inside the host. Therefore we analysed the *in vivo* expression database to get useful information on the questions if *E*. *faecalis* is metabolically active and which substrates are used by this bacterium during the first hours of infection.

**Fig 2:**
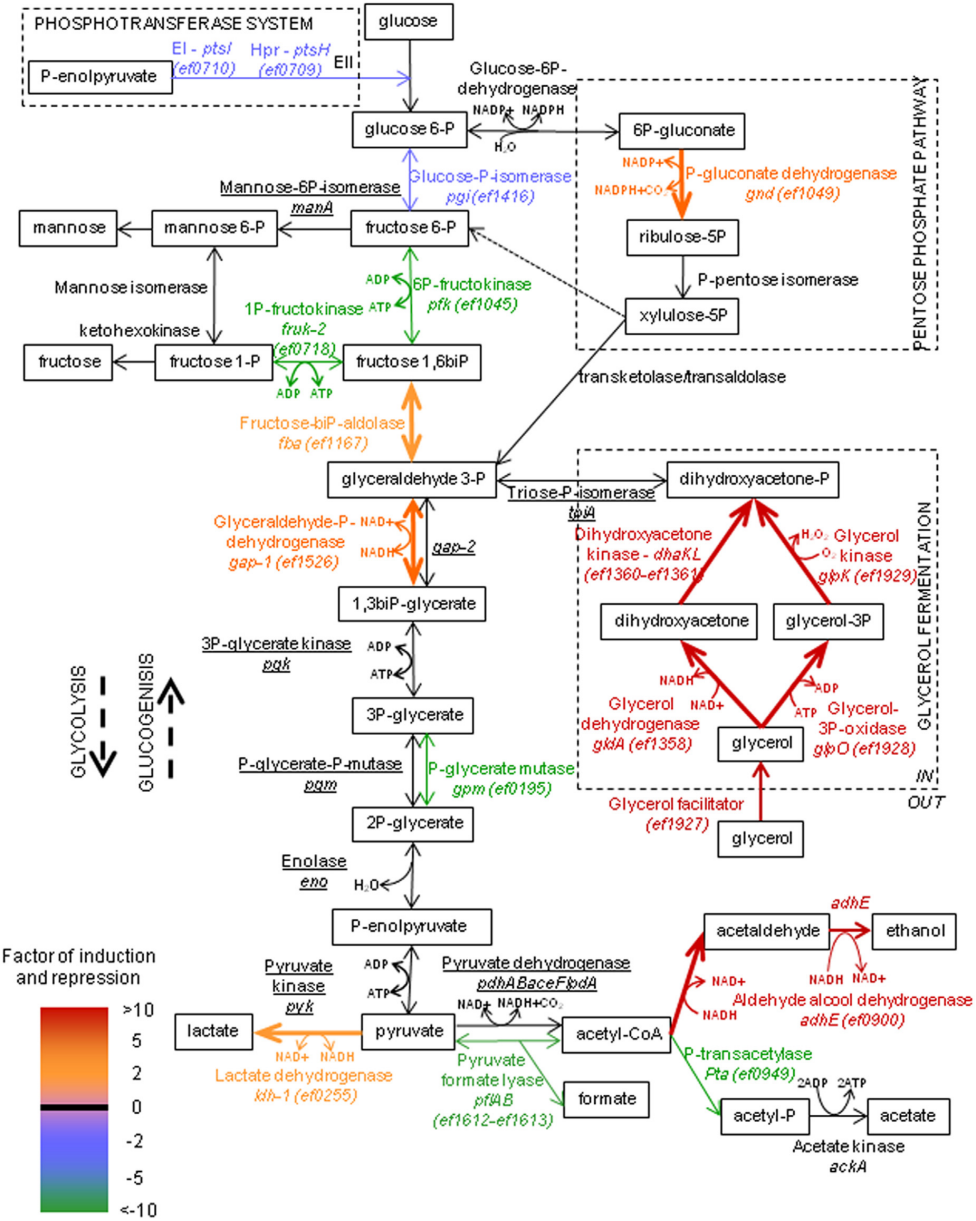
Expression of central metabolic pathways of *E*. *faecalis* during intraperitoneal infection. RNA-Seq data from *in vivo* conditions are compared to mid-log and stationary growth phase cultures in BHI. Genes involved in glycolysis, gluconeogenesis, pentose phosphate pathways and fermentations with their respective transcriptional activities are shown, according to the indicated colour scale. No change is indicated in black, and genes significantly expressed are underlined (>500 reads).

The data indicate that genes belonging to the category “Metabolism” are part of the second most abundant group of genes (18%) arguing that the bacteria actively adapt to the special nutrient conditions inside the host. We noticed that the general phosphotransferase system (PTS) proteins HPr and EI (EF0709 and EF0710, respectively) are significantly downregulated (-4.9-fold) as well as a fructose specific PTS (EF0717; -15.7-fold) (S4 Table and Fig 2). This suggests that PTS sugars may be scarce in the mouse peritoneum, a conclusion supported by the fact that expression of only 2 genes out of 88 genes encoding PTS components (including 35 PTS sugar transporters [59]) are induced *in vivo*. One of them, encoding an orphan enzyme IIB, is moderately induced (*ef1769*; +3.8 fold) but the second gene encoding a PTS IIC component is more strongly upregulated inside the mouse peritoneum (*ef0292*; +37.0-fold). This last gene is probably in an operon with gene *ef0291* (S1 Fig) encoding a 6-phospho-β-glucosidase which suggests that the PTS IIC component is likely involved in the transport of β-glucosides. Three other genes collectively encoding an ABC transport system are highly induced *in vivo* (*ef1343-ef1345*; +15.4-fold). This system demonstrates significant homologies to maltodextrin-specific ABC transporters of other Gram-positive bacteria [60,61] including *E*. *faecium* [62]. This suggests that maltodextrines may also be available for the bacterium inside the host, *i*.*e*. from glycogen breakdown.

Other glycopolymers available to the bacteria during mouse peritonitis may be chitin-like polysaccharides. An operon structure (*ef0361-ef0362*) encoding a chitinase and a chitin-binding protein, has a strongly induced expression (+46.3-fold). Chitin is a β-glucoside composed of N-acetyl-glucosamine residues which differs from cellulose only by the acetamid group. Therefore, it can be tentatively supposed that the concomitant induction of the above mentioned operon (*ef0291-0292*) encoding a 6-phospho-β-glucosidase as well as a PTS IIC component may be functionally linked. It has been shown that the chitin-binding protein of *E*. *faecalis* cleaves chitin in an oxidative reaction, and acts synergistically with the chitinase [63]. Recent evidence indicates that bacterial chitinases and chitin-binding proteins contribute to bacterial adherence to chitin-like molecules present on the surface of mammalian cells and it has been demonstrated that *Listeria monocytogenes*-secreted chitinase suppress host innate immunity [64].

After transport and if necessary hydrolytic cleavage, the monomeric sugars will enter one of the three pathways present in *E*. *faecalis*, glycolysis, pentose phosphate or Entner-Duderoff pathway (Fig 2). Our data indicate that the upper part of glycolysis seems to be downregulated due to the reduced expression of the genes encoding 6-phosphofructokinase (*ef1045;* -11.6-fold) and, to a lesser extend, glucose-6-phosphate isomerase (*ef1416*; -3.1-fold). On the other hand, 6-phosphogluconate dehydrogenase of the Pentose Phosphate Pathway (PPP) is strongly induced (*ef1049*; +5.5-fold) suggesting that sugars entering into glycolysis are, at least partly, shifted into the PPP through reduction of glucose-6-phosphate to 6-phosphogluconate. One of the key functions of the PPP is the synthesis of reducing power in form of NADPH, which is a cofactor for numerous biosynthetic reactions. So, the shift of the carbon flow into this pathway may reflect an increased need of the cells for biosynthesis of essential precursors not present at the side of infection in comparison to the conditions of a rich laboratory medium.

Carbon flow from the upper to the lower part of glycolysis as well as the flux through the lower part seems to be stimulated since expression of fructose-1,6-bisphosphate aldolase gene (*ef1167*; +3.6-fold) and one out of two genes encoding glyceraldehyde-3-phosphate dehydrogenases (*ef1526*; +3.8-fold) are induced inside the mouse peritoneum. Of note, one gene out of five paralogues encoding phosphoglycerate mutases was strongly repressed under *in vivo* conditions (*ef0195*; -23.9-fold).

Apart from sugars, glycerol seems to be an important substrate for *E*. *faecalis in vivo*. Two pathways for glycerol dissimilation are present in this bacterium (Fig 2) and the corresponding genes are grouped in two operon structures (*ef1358-ef1361* and *ef1927-1929*) [65]. In the dihydroxyacetone (DHA) pathway (*ef1358-ef1361*), glycerol is first oxidized to DHA by glycerol dehydrogenase GldA and then phosphorylated to DHAP by DHA kinase DhaKL. In the GlpK pathway (*ef1927-1929*), glycerol is first phosphorylated to glycerol-3-phosphate by glycerol kinase GlpK and then oxidized to DHA-P by glycerol-3-phosphate oxidase GlpO [65]. Both operon structures have a strongly induced expression during mice peritonitis (+6.4-fold and +12.3-fold, respectively). Interestingly, glycerol has been identified as an important substrate for *E*. *faecalis* adaptive fitness in mouse intestinal tract [66], and also for other bacterial pathogens, especially *L*. *monocytogenes* [17,67,68]. This bacterium is a facultative intracellular pathogen infecting several different vertebrate and invertebrate cell types including macrophages. After phagocytosis, *L*. *monocytogenes* escapes from the phagolysosome and freely replicates in the host cytoplasm. Several independent studies using transcriptomic and metabolomic approaches demonstrated that glycerol is used inside cells for proliferation [67]. This seems also to be the case for *Shigella flexneri*, another intracellular pathogen replicating in the cytoplasm of infected cells but not for *Mycobacterium tuberculosis* and *Salmonella typhimurium* which replicate inside the phagolysosomes [67]. From these combined data it can be suggested that *E*. *faecalis*, like *L*. *monocytogenes*, might be able to escape from the phagolysosomes and replicate in the cytoplasm by using glycerol as a substrate.

Concerning final fermentation end products, lactate and ethanol seem to be formed *in vivo*. Expression of pyruvate formate lyase is very strongly repressed (*ef1612*, -22.9-fold), which might indicate that acetylCoA is synthezised mainly by pyruvate dehydrogenase. In this reaction additional reducing power in the form of NADH is generated. The acetylCoA formed can be converted to acetate which is energetically interesting for the cells since this leads to the generation of one additional ATP. However, the expression of the gene encoding phosphotransacetylase (*ef0949*) catalysing the first reaction of this path is strongly repressed *in vivo* (-48.7-fold). Furthermore, the gene encoding the bi-functional aldehyde-alcool dehydrogenase (*ef0900*) catalysing the reduction of acetylCoA to ethanol, has a significantly induced expression (+11.7-fold). Therefore, acetylCoA seems to be mainly (if not exclusively) converted to ethanol under peritonitis infection conditions. Part of the pyruvate formed is probably also reduced to lactate since the gene encoding the main lactate dehydrogenase (*ef0255*) [69] is somewhat induced (+3.0 fold) in cells infecting the mouse peritoneum.

### Comparison of the data to previous *in vivo* transcriptome studies

We present in this work the first genome-wide analysis of the *E*. *faecalis* transcriptome during peritonitis by high-resolution RNA-Sequencing. Very recently, a microarray-based transcriptome of *E*. *faecalis* was conducted with bacteria isolated from a rabbit subdermal abscess infection site [70]. These analyses revealed that at 2 hours post-inoculation of the subdemal chambers, 222 genes were differentially expressed, with around half of them induced when compared with the initial inoculum. Comparison with our database revealed only 9% overlap with genes differentially expressed in the rabit model. At 8 hours post inoculation of the subdermal chambers, 88% of the differentially expressed genes (total number of 291 genes) were repressed. Concerning the genes induced in the rabbit model, 19 overlap with our database of *in vivo* induced genes, amongst others *ef0362* encoding the chitin-binding protein, *ef0463* encoding the superoxide dismutase, genes encoding stress response proteins like *ef1058*, *lpB*, *groEL* and *groES*, and 3 lipoproteins (*ef0095*, *ef0176*, *ef0177*). The differences of the animal models, sites of infection, time point of sampling, and the techniques used for the transcriptome analysis could be at the basis of this low overlap between the two datasets. It is of note that bacteria incubated in the subdermal chambers in the rabbit abscess infection model do not survive well in the first hours of incubation with around 10% and 4% of the bacteria still culturable at 2 hours and 8 hours post-inoculation, in contrast to the peritonitis model used in the present study (S2 Fig).

Before the introduction of microarray and RNA-Seq technologies, identification of upregulated genes has been performed by a recombinant-based *in vivo* expression technology (RIVET) approach. However, using the above mentioned rabbit subdermal abcess infection model, only very few RIVET clones overlapped with the microarray results. We used the RIVET for the identification of genes activated during bacteremia and peritonitis infections in *E*. *faecalis* [71]. 20 genes grouped in 7 operon structures identified to be induced *in vivo* in the present study overlap with the 64 genes identified by RIVET (*ef0104* to *ef0108*, *ef0162* to *ef0164*, *ef0176-ef0177*, *ef0185* to *ef0187*, *ef0377*-*ef0378*, *ef0525* to *ef0528*, and *ef1677*). The identification of the *in vivo* induction of these genes by two different techniques may indicate that they should be particularly important for the virulence process in *E*. *faecalis*.

### Mutants affected in genes highly induced *in vivo* should be attenuated in virulence

**Fig 3:**
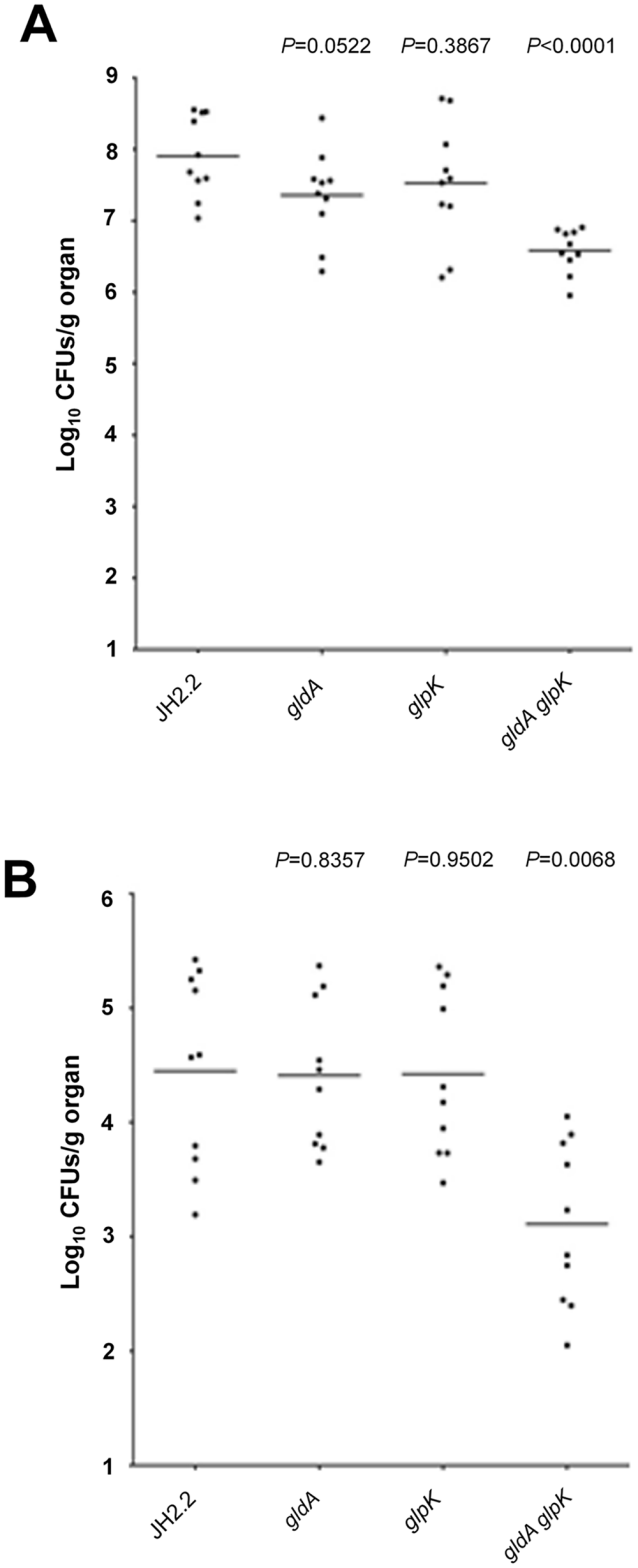
Bacterial persistence within mouse organs. Enterococcal tissue burdens in kidneys and in livers from BALB/c mice infected intravenously with 1 × 10^8^ cells of *E*. *faecalis* wild-type JH2-2 and the *ΔgldA*, *ΔglpK*, and *ΔgldA/glpK* mutant strains are shown. Kidney pair (A) and liver (B) homogenates were obtained from groups of 10 mice sacrificed and necropsied at day 7 postinfection. The results, expressed as log_10_ CFU per gram of tissue, represent values recorded separately for each of the 10 mice. Horizontal bars represent the geometric means. *P* values of less than 0.05 were considered to be significant.

Our *in vivo* expression database identified several operons which are highly induced during peritonitis and hence should be important for the infection process. To verify this assumption, we tested previously well characterized mutants affected in glycerol metabolism [65] in a systemic murine infection model. Seven days after the intravenous bacterial challenge, groups of mice were sacrificed, and kidneys and livers removed to evaluate the presence of viable bacteria. As shown in Fig 3, the bacterial counts, in terms of CFU per gram of tissue, determined from the infected organs were significantly different only between the *gldA/glpK* double mutant and the parent strain JH2-2. In fact, the numbers of recovered cells from the *gldA*/*glpK* deficient strain were significantly lower than those of the wild-type strains in both kidneys (*P <*0.0001, Fig 3A) and livers (*P =* 0.0068, Fig 3B), with consistent reductions observed (1.37 log_10_ units in the kidneys and 1.42 log_10_ units in the liver). These findings indicate that the ability to metabolize glycerol contributes to the enterococcal colonisation within the mouse organs and support both our database and the assumption that highly-induced *in vivo* genes should be important for the virulence of *E*. *faecalis*. Thus, this comprehensive database contributes towards a deeper understanding of the opportunistic behaviour of *E*. *faecalis* during infection.

## Supporting Information

S1 Fig Genomic organizations of the CDS of *E*. *faecalis* V583 with induced or repressed expression during mice peritonitis. CDS with induced (A-B) or repressed (C-D) expression *in vivo* are represented (gray arrows) with their previous and following genes (white arrows). If present, terminators are represented. Distances between genes are indicated in bp under the respective intergenic regions and negative digits indicate overlap of the adjacent CDS. (A and C) List of *in vivo* induced and repressed CDS with deduced or experimentally proven operon structure (citations indicated). CDS with no ambiguity concerning their operon structure are listed, for *in vivo* induced and repressed genes, respectively. (B and D) RT-PCR assays were systematically performed when no experimental evidence of the operon structure was found in the literature and for flanking genes at a distance below 200 pb for *in vivo* induced and repressed genes, respectively. Triangles represent forward (in white) and reverse (in black) primers used for RT-PCR experiments to check operon structures, and amplified PCR products of 290 to 310 bp length. Line next to the ladder indicates the expected size of the PCR product.
(PDF)

S2 Fig Survival of *E*. *faecalis* in the mouse peritoneum. Mice were infected intraperitoneally with 2.10^8^ CFU. The bacterial load was quantified by plating peritoneum wash sample dilutions in PBS onto BHI agar. The t0 time point represents the *E*. *faecalis* recovery 15 min after inoculation. The values represent the average of three biological replicates and error bars are indicated for each time point.
(PDF)

S1 Table List of CDS of *E*. *faecalis* V583 considered for expression analysis. (XLSX)

S2 Table RT-PCR primers. (XLSX)

S3 Table List of *in vivo* induced genes in *E*. *faecalis* V583 during peritonitis infection. The *in vivo* induced genes are indicated with their factors of induction calculated for intraperitoneal infection of mice compared to mid-log phase or 24h culture in BHI. The factors of induction obtained with PCRq experiments are also indicated. Parts in the table highlighted in grey correspond to genes belonging to the pathogenicity island (*ef0479-ef0628*). (_a_) Mean fold induction is the factor calculated as the average of all the factors available for each operon, mentioned in the text.
(XLSX)

S4 Table List of *in vivo* repressed genes in *E*. *faecalis* V583 during peritonitis infection. The *in vivo* repressed genes are listed with their factors of repression calculated for intraperitoneal infection of mice compared to mid-log phase or 24h culture in BHI. (_a_) Mean fold induction is the factor calculated as the average of all the factors available for each operon, mentioned in the text.
(XLSX)

S5 Table qPCR primers. (XLSX)

## References

[1] Zarb P, Coignard B, Griskeviciene J, Muller A, Vankerckhoven V, Weist K, et al The European Centre for Disease Prevention and Control (ECDC) pilot point prevalence survey of healthcare-associated infections and antimicrobial use. Euro Surveill Bull Eur Sur Mal Transm Eur Commun Dis Bull. 2012;17. 10.2807/ese.17.46.20316-en 23171822

[2] Sievert DM, Ricks P, Edwards JR, Schneider A, Patel J, Srinivasan A, et al Antimicrobial-resistant pathogens associated with healthcare-associated infections: summary of data reported to the National Healthcare Safety Network at the Centers for Disease Control and Prevention, 2009–2010. Infect Control Hosp Epidemiol Off J Soc Hosp Epidemiol Am. 2013;34: 1–14. 10.1086/668770 23221186

[3] Werner G, Coque TM, Hammerum AM, Hope R, Hryniewicz W, Johnson A, et al Emergence and spread of vancomycin resistance among enterococci in Europe. Euro Surveill Bull Eur Sur Mal Transm Eur Commun Dis Bull. 2008;13. 19021959

[4] Rincé A, Le Breton Y, Verneuil N, Giard J-C, Hartke A, Auffray Y. Physiological and molecular aspects of bile salt response in Enterococcus faecalis. Int J Food Microbiol. 2003;88: 207–213. 14596992 10.1016/s0168-1605(03)00182-x

[5] Sherman JM. The Enterococci and Related Streptococci. J Bacteriol. 1938;35: 81–93. 16560094 10.1128/jb.35.2.81-93.1938 PMC374429

[6] Kramer A, Schwebke I, Kampf G. How long do nosocomial pathogens persist on inanimate surfaces? A systematic review. BMC Infect Dis. 2006;6: 130 10.1186/1471-2334-6-130 16914034 PMC1564025

[7] Giard JC, Hartke A, Flahaut S, Benachour A, Boutibonnes P, Auffray Y. Starvation-induced multiresistance in Enterococcus faecalis JH2-2. Curr Microbiol. 1996;32: 264–271. 8857273 10.1007/s002849900048

[8] Garsin DA, Frank KL, Silanpää J, Ausubel FM, Hartke A, Shankar N, et al Pathogenesis and Models of Enterococcal Infection In: Gilmore MS, Clewell DB, Ike Y, Shankar N, editors. Enterococci: From Commensals to Leading Causes of Drug Resistant Infection. Boston: Massachusetts Eye and Ear Infirmary; 2014 Available: http://www.ncbi.nlm.nih.gov/books/NBK190426/ 24649512

[9] Shankar N, Baghdayan AS, Gilmore MS. Modulation of virulence within a pathogenicity island in vancomycin-resistant Enterococcus faecalis. Nature. 2002;417: 746–750. 10.1038/nature00802 12066186

[10] Coburn PS, Baghdayan AS, Dolan GT, Shankar N. An AraC-type transcriptional regulator encoded on the Enterococcus faecalis pathogenicity island contributes to pathogenesis and intracellular macrophage survival. Infect Immun. 2008;76: 5668–5676. 10.1128/IAI.00930-08 18824537 PMC2583563

[11] Febrer M, McLay K, Caccamo M, Twomey KB, Ryan RP. Advances in bacterial transcriptome and transposon insertion-site profiling using second-generation sequencing. Trends Biotechnol. 2011;29: 586–594. 10.1016/j.tibtech.2011.06.004 21764162

[12] Wang Z, Gerstein M, Snyder M. RNA-Seq: a revolutionary tool for transcriptomics. Nat Rev Genet. 2009;10: 57–63. 10.1038/nrg2484 19015660 PMC2949280

[13] Jorth P, Trivedi U, Rumbaugh K, Whiteley M. Probing bacterial metabolism during infection using high-resolution transcriptomics. J Bacteriol. 2013; 10.1128/JB.00875-13 PMC3811578 23974023

[14] Yoder-Himes DR, Chain PSG, Zhu Y, Wurtzel O, Rubin EM, Tiedje JM, et al Mapping the Burkholderia cenocepacia niche response via high-throughput sequencing. Proc Natl Acad Sci U S A. 2009;106: 3976–3981. 10.1073/pnas.0813403106 19234113 PMC2645912

[15] Sharma CM, Hoffmann S, Darfeuille F, Reignier J, Findeiss S, Sittka A, et al The primary transcriptome of the major human pathogen Helicobacter pylori. Nature. 2010;464: 250–255. 10.1038/nature08756 20164839

[16] Albrecht M, Sharma CM, Reinhardt R, Vogel J, Rudel T. Deep sequencing-based discovery of the Chlamydia trachomatis transcriptome. Nucleic Acids Res. 2010;38: 868–877. 10.1093/nar/gkp1032 19923228 PMC2817459

[17] Mandlik A, Livny J, Robins WP, Ritchie JM, Mekalanos JJ, Waldor MK. RNA-Seq-Based Monitoring of Infection-Linked Changes in Vibrio cholerae Gene Expression. Cell Host Microbe. 2011;10: 165–174. 10.1016/j.chom.2011.07.007 21843873 PMC3166260

[18] Taveirne ME, Theriot CM, Livny J, DiRita VJ. The complete Campylobacter jejuni transcriptome during colonization of a natural host determined by RNAseq. PloS One. 2013;8: e73586 10.1371/journal.pone.0073586 23991199 PMC3749233

[19] Skvortsov TA, Ignatov DV, Majorov KB, Apt AS, Azhikina TL. Mycobacterium tuberculosis Transcriptome Profiling in Mice with Genetically Different Susceptibility to Tuberculosis. Acta Naturae. 2013;5: 62–69. 23819037 PMC3695354

[20] Hannon G. FASTX toolkit [Internet]. Available: http://hannonlab.cshl.edu/fastx_toolkit/

[21] Martin M. Cutadapt removes adapter sequences from high-throughput sequencing reads. EMBnet.journal. 2011;17: pp. 10–12. 10.14806/ej.17.1.200

[22] Rice P, Longden I, Bleasby A. EMBOSS: the European Molecular Biology Open Software Suite. Trends Genet TIG. 2000;16: 276–277. 10827456 10.1016/s0168-9525(00)02024-2

[23] Li H, Durbin R. Fast and accurate long-read alignment with Burrows-Wheeler transform. Bioinforma Oxf Engl. 2010;26: 589–595. 10.1093/bioinformatics/btp698 PMC2828108 20080505

[24] Li H, Handsaker B, Wysoker A, Fennell T, Ruan J, Homer N, et al The Sequence Alignment/Map format and SAMtools. Bioinforma Oxf Engl. 2009;25: 2078–2079. 10.1093/bioinformatics/btp352 PMC2723002 19505943

[25] Zhang Y, Liu T, Meyer CA, Eeckhoute J, Johnson DS, Bernstein BE, et al Model-based analysis of ChIP-Seq (MACS). Genome Biol. 2008;9: R137 10.1186/gb-2008-9-9-r137 18798982 PMC2592715

[26] Robinson MD, Oshlack A. A scaling normalization method for differential expression analysis of RNA-seq data. Genome Biol. 2010;11: R25 10.1186/gb-2010-11-3-r25 20196867 PMC2864565

[27] Tarazona S, García-Alcalde F, Dopazo J, Ferrer A, Conesa A. Differential expression in RNA-seq: a matter of depth. Genome Res. 2011;21: 2213–2223. 10.1101/gr.124321.111 21903743 PMC3227109

[28] Robinson JT, Thorvaldsdóttir H, Winckler W, Guttman M, Lander ES, Getz G, et al Integrative genomics viewer. Nat Biotechnol. 2011;29: 24–26. 10.1038/nbt.1754 21221095 PMC3346182

[29] Thorvaldsdóttir H, Robinson JT, Mesirov JP. Integrative Genomics Viewer (IGV): high-performance genomics data visualization and exploration. Brief Bioinform. 2013;14: 178–192. 10.1093/bib/bbs017 22517427 PMC3603213

[30] Coburn PS, Gilmore MS. The Enterococcus faecalis cytolysin: a novel toxin active against eukaryotic and prokaryotic cells. Cell Microbiol. 2003;5: 661–669. 12969372 10.1046/j.1462-5822.2003.00310.x

[31] Van Tyne D, Martin MJ, Gilmore MS. Structure, function, and biology of the Enterococcus faecalis cytolysin. Toxins. 2013;5: 895–911. 10.3390/toxins5050895 23628786 PMC3709268

[32] Lowe AM, Lambert PA, Smith AW. Cloning of an Enterococcus faecalis endocarditis antigen: homology with adhesins from some oral streptococci. Infect Immun. 1995;63: 703–706. 7822045 10.1128/iai.63.2.703-706.1995 PMC173055

[33] Low YL, Jakubovics NS, Flatman JC, Jenkinson HF, Smith AW. Manganese-dependent regulation of the endocarditis-associated virulence factor EfaA of Enterococcus faecalis. J Med Microbiol. 2003;52: 113–119. 12543916 10.1099/jmm.0.05039-0

[34] Singh KV, Qin X, Weinstock GM, Murray BE. Generation and testing of mutants of Enterococcus faecalis in a mouse peritonitis model. J Infect Dis. 1998;178: 1416–1420. 9780263 10.1086/314453

[35] Qin X, Singh KV, Weinstock GM, Murray BE. Effects of Enterococcus faecalis fsr genes on production of gelatinase and a serine protease and virulence. Infect Immun. 2000;68: 2579–2586. 10768947 10.1128/iai.68.5.2579-2586.2000 PMC97462

[36] Podbielski A, Kreikemeyer B. Cell density—dependent regulation: basic principles and effects on the virulence of Gram-positive cocci. Int J Infect Dis IJID Off Publ Int Soc Infect Dis. 2004;8: 81–95. 10.1016/j.ijid.2003.04.003 14732326

[37] Pinkston KL, Gao P, Diaz-Garcia D, Sillanpää J, Nallapareddy SR, Murray BE, et al The Fsr quorum-sensing system of Enterococcus faecalis modulates surface display of the collagen-binding MSCRAMM Ace through regulation of gelE. J Bacteriol. 2011;193: 4317–4325. 10.1128/JB.05026-11 21705589 PMC3165527

[38] Singh KV, Nallapareddy SR, Sillanpää J, Murray BE. Importance of the collagen adhesin ace in pathogenesis and protection against Enterococcus faecalis experimental endocarditis. PLoS Pathog. 2010;6: e1000716 10.1371/journal.ppat.1000716 20072611 PMC2798748

[39] Hendrickx APA, Willems RJL, Bonten MJM, van Schaik W. LPxTG surface proteins of enterococci. Trends Microbiol. 2009;17: 423–430. 10.1016/j.tim.2009.06.004 19726195

[40] Chow JW, Thal LA, Perri MB, Vazquez JA, Donabedian SM, Clewell DB, et al Plasmid-associated hemolysin and aggregation substance production contribute to virulence in experimental enterococcal endocarditis. Antimicrob Agents Chemother. 1993;37: 2474–2477. 8285637 10.1128/aac.37.11.2474 PMC192412

[41] Dupont H, Montravers P, Mohler J, Carbon C. Disparate findings on the role of virulence factors of Enterococcus faecalis in mouse and rat models of peritonitis. Infect Immun. 1998;66: 2570–2575. 9596718 10.1128/iai.66.6.2570-2575.1998 PMC108240

[42] Leendertse M, Willems RJL, Giebelen IAJ, Roelofs JJTH, Bonten MJM, van der Poll T. Neutrophils are essential for rapid clearance of Enterococcus faecium in mice. Infect Immun. 2009;77: 485–491. 10.1128/IAI.00863-08 19001080 PMC2612258

[43] Rincé A, Giard JC, Pichereau V, Flahaut S, Auffray Y. Identification and characterization of gsp65, an organic hydroperoxide resistance (ohr) gene encoding a general stress protein in Enterococcus faecalis. J Bacteriol. 2001;183: 1482–1488. 10.1128/JB.183.4.1482-1488.2001 11157966 PMC95027

[44] Rincé A, Uguen M, Le Breton Y, Giard J-C, Flahaut S, Dufour A, et al The Enterococcus faecalis gene encoding the novel general stress protein Gsp62. Microbiol Read Engl. 2002;148: 703–711. 10.1099/00221287-148-3-703 11882704

[45] Chiancone E, Ceci P, Ilari A, Ribacchi F, Stefanini S. Iron and proteins for iron storage and detoxification. Biometals Int J Role Met Ions Biol Biochem Med. 2004;17: 197–202. 10.1023/b:biom.0000027692.24395.76 15222465

[46] Deuerling E, Patzelt H, Vorderwülbecke S, Rauch T, Kramer G, Schaffitzel E, et al Trigger Factor and DnaK possess overlapping substrate pools and binding specificities. Mol Microbiol. 2003;47: 1317–1328. 12603737 10.1046/j.1365-2958.2003.03370.x

[47] De Oliveira NEM, Abranches J, Gaca AO, Laport MS, Damaso CR, Bastos M do C de F, et al clpB, a class III heat-shock gene regulated by CtsR, is involved in thermotolerance and virulence of Enterococcus faecalis. Microbiol Read Engl. 2011;157: 656–665. 10.1099/mic.0.041897-0 PMC5974289 21148206

[48] Verneuil N, Rincé A, Sanguinetti M, Auffray Y, Hartke A, Giard J-C. Implication of hypR in the virulence and oxidative stress response of Enterococcus faecalis. FEMS Microbiol Lett. 2005;252: 137–141. 10.1016/j.femsle.2005.08.043 16216443

[49] La Carbona S, Sauvageot N, Giard J-C, Benachour A, Posteraro B, Auffray Y, et al Comparative study of the physiological roles of three peroxidases (NADH peroxidase, Alkyl hydroperoxide reductase and Thiol peroxidase) in oxidative stress response, survival inside macrophages and virulence of Enterococcus faecalis. Mol Microbiol. 2007;66: 1148–1163. 10.1111/j.1365-2958.2007.05987.x 17971082

[50] Verneuil N, Mazé A, Sanguinetti M, Laplace J-M, Benachour A, Auffray Y, et al Implication of (Mn)superoxide dismutase of Enterococcus faecalis in oxidative stress responses and survival inside macrophages. Microbiol Read Engl. 2006;152: 2579–2589. 10.1099/mic.0.28922-0 16946253

[51] Frankenberg L, Brugna M, Hederstedt L. Enterococcus faecalis heme-dependent catalase. J Bacteriol. 2002;184: 6351–6356. 12399505 10.1128/JB.184.22.6351-6356.2002 PMC151946

[52] Verneuil N, Rincé A, Sanguinetti M, Posteraro B, Fadda G, Auffray Y, et al Contribution of a PerR-like regulator to the oxidative-stress response and virulence of Enterococcus faecalis. Microbiol Read Engl. 2005;151: 3997–4004. 10.1099/mic.0.28325-0 16339944

[53] Rochat T, Boudebbouze S, Gratadoux J-J, Blugeon S, Gaudu P, Langella P, et al Proteomic analysis of spontaneous mutants of Lactococcus lactis: Involvement of GAPDH and arginine deiminase pathway in H2O2 resistance. Proteomics. 2012;12: 1792–1805. 10.1002/pmic.201100465 22623348

[54] Roche B, Aussel L, Ezraty B, Mandin P, Py B, Barras F. Iron/sulfur proteins biogenesis in prokaryotes: formation, regulation and diversity. Biochim Biophys Acta. 2013;1827: 455–469. 10.1016/j.bbabio.2012.12.010 23298813

[55] Riboldi GP, de Oliveira JS, Frazzon J. Enterococcus faecalis SufU scaffold protein enhances SufS desulfurase activity by acquiring sulfur from its cysteine-153. Biochim Biophys Acta. 2011;1814: 1910–1918. 10.1016/j.bbapap.2011.06.016 21835272

[56] Huet G, Daffé M, Saves I. Identification of the Mycobacterium tuberculosis SUF machinery as the exclusive mycobacterial system of [Fe-S] cluster assembly: evidence for its implication in the pathogen’s survival. J Bacteriol. 2005;187: 6137–6146. 10.1128/JB.187.17.6137-6146.2005 16109955 PMC1196142

[57] Matos RC, Lapaque N, Rigottier-Gois L, Debarbieux L, Meylheuc T, Gonzalez-Zorn B, et al Enterococcus faecalis prophage dynamics and contributions to pathogenic traits. PLoS Genet. 2013;9: e1003539 10.1371/journal.pgen.1003539 23754962 PMC3675006

[58] Nallapareddy SR, Wenxiang H, Weinstock GM, Murray BE. Molecular characterization of a widespread, pathogenic, and antibiotic resistance-receptive Enterococcus faecalis lineage and dissemination of its putative pathogenicity island. J Bacteriol. 2005;187: 5709–5718. 10.1128/JB.187.16.5709-5718.2005 16077117 PMC1196071

[59] Gilmore MS, Lebreton F, van Schaik W. Genomic transition of enterococci from gut commensals to leading causes of multidrug-resistant hospital infection in the antibiotic era. Curr Opin Microbiol. 2013;16: 10–16. 10.1016/j.mib.2013.01.006 23395351 PMC3649759

[60] Schönert S, Seitz S, Krafft H, Feuerbaum E-A, Andernach I, Witz G, et al Maltose and maltodextrin utilization by Bacillus subtilis. J Bacteriol. 2006;188: 3911–3922. 10.1128/JB.00213-06 16707683 PMC1482931

[61] Gopal S, Berg D, Hagen N, Schriefer E-M, Stoll R, Goebel W, et al Maltose and maltodextrin utilization by Listeria monocytogenes depend on an inducible ABC transporter which is repressed by glucose. PloS One. 2010;5: e10349 10.1371/journal.pone.0010349 20436965 PMC2860498

[62] Zhang X, Rogers M, Bierschenk D, Bonten MJM, Willems RJL, van Schaik W. A LacI-family regulator activates maltodextrin metabolism of Enterococcus faecium. PloS One. 2013;8: e72285 10.1371/journal.pone.0072285 23951303 PMC3737153

[63] Vaaje-Kolstad G, Bøhle LA, Gåseidnes S, Dalhus B, Bjørås M, Mathiesen G, et al Characterization of the chitinolytic machinery of Enterococcus faecalis V583 and high-resolution structure of its oxidative CBM33 enzyme. J Mol Biol. 2012;416: 239–254. 10.1016/j.jmb.2011.12.033 22210154

[64] Chaudhuri S, Gantner BN, Ye RD, Cianciotto NP, Freitag NE. The Listeria monocytogenes ChiA chitinase enhances virulence through suppression of host innate immunity. mBio. 2013;4: e00617–00612. 10.1128/mBio.00617-12 23512964 PMC3604766

[65] Bizzini A, Zhao C, Budin-Verneuil A, Sauvageot N, Giard J-C, Auffray Y, et al Glycerol is metabolized in a complex and strain-dependent manner in Enterococcus faecalis. J Bacteriol. 2010;192: 779–785. 10.1128/JB.00959-09 19966010 PMC2812454

[66] Lindenstrauss AG, Ehrmann MA, Behr J, Landstorfer R, Haller D, Sartor RB, et al Transcriptome analysis of Enterococcus faecalis toward its adaption to surviving in the mouse intestinal tract. Arch Microbiol. 2014;196: 423–433. 10.1007/s00203-014-0982-2 24700373

[67] Eisenreich W, Dandekar T, Heesemann J, Goebel W. Carbon metabolism of intracellular bacterial pathogens and possible links to virulence. Nat Rev Microbiol. 2010;8: 401–412. 10.1038/nrmicro2351 20453875

[68] Joseph B, Przybilla K, Stühler C, Schauer K, Slaghuis J, Fuchs TM, et al Identification of Listeria monocytogenes genes contributing to intracellular replication by expression profiling and mutant screening. J Bacteriol. 2006;188: 556–568. 10.1128/JB.188.2.556-568.2006 16385046 PMC1347271

[69] Rana NF, Sauvageot N, Laplace J-M, Bao Y, Nes I, Rincé A, et al Redox balance via lactate dehydrogenase is important for multiple stress resistance and virulence in Enterococcus faecalis. Infect Immun. 2013;81: 2662–2668. 10.1128/IAI.01299-12 23649090 PMC3719593

[70] Frank KL, Colomer-Winter C, Grindle SM, Lemos JA, Schlievert PM, Dunny GM. Transcriptome Analysis of Enterococcus faecalis during Mammalian Infection Shows Cells Undergo Adaptation and Exist in a Stringent Response State. PloS One. 2014;9: e115839 10.1371/journal.pone.0115839 25545155 PMC4278851

[71] Hanin A, Sava I, Bao Y, Huebner J, Hartke A, Auffray Y, et al Screening of in vivo activated genes in Enterococcus faecalis during insect and mouse infections and growth in urine. PloS One. 2010;5: e11879 10.1371/journal.pone.0011879 20686694 PMC2912369

