# Supplementary material for: The Intraperitoneal Transcriptome of the Opportunistic Pathogen Enterococcus faecalis in Mice
Source: PLoS One. 2015 May 15;10(5):e0126143. doi: 10.1371/journal.pone.0126143 (PMC4433114; doi:10.1371/journal.pone.0126143)
Supplement: S1 Fig — CDS with induced (A-B) or repressed (C-D) expression in vivo are represented (gray arrows) with their previous and following genes (white arrows). If present, terminators are represented. Distances between genes are indicated in bp under the respective intergenic regions and negative digits indicate overlap of the adjacent CDS. (A and C) List of in vivo induced and repressed CDS with deduced or experimentally proven operon structure (citations indicated). CDS with no ambiguity concerning their operon structure are listed, for in vivo induced and repressed genes, respectively. (B and D) RT-PCR assays were systematically performed when no experimental evidence of the operon structure was found in the literature and for flanking genes at a distance below 200 pb for in vivo induced and repressed genes, respectively. Triangles represent forward (in white) and reverse (in black) primers used for RT-PCR experiments to check operon structures, and amplified PCR products of 290 to 310 bp length. Line next to the ladder indicates the expected size of the PCR product. (PDF) [file pone.0126143.s001.pdf]

Figure S1-A

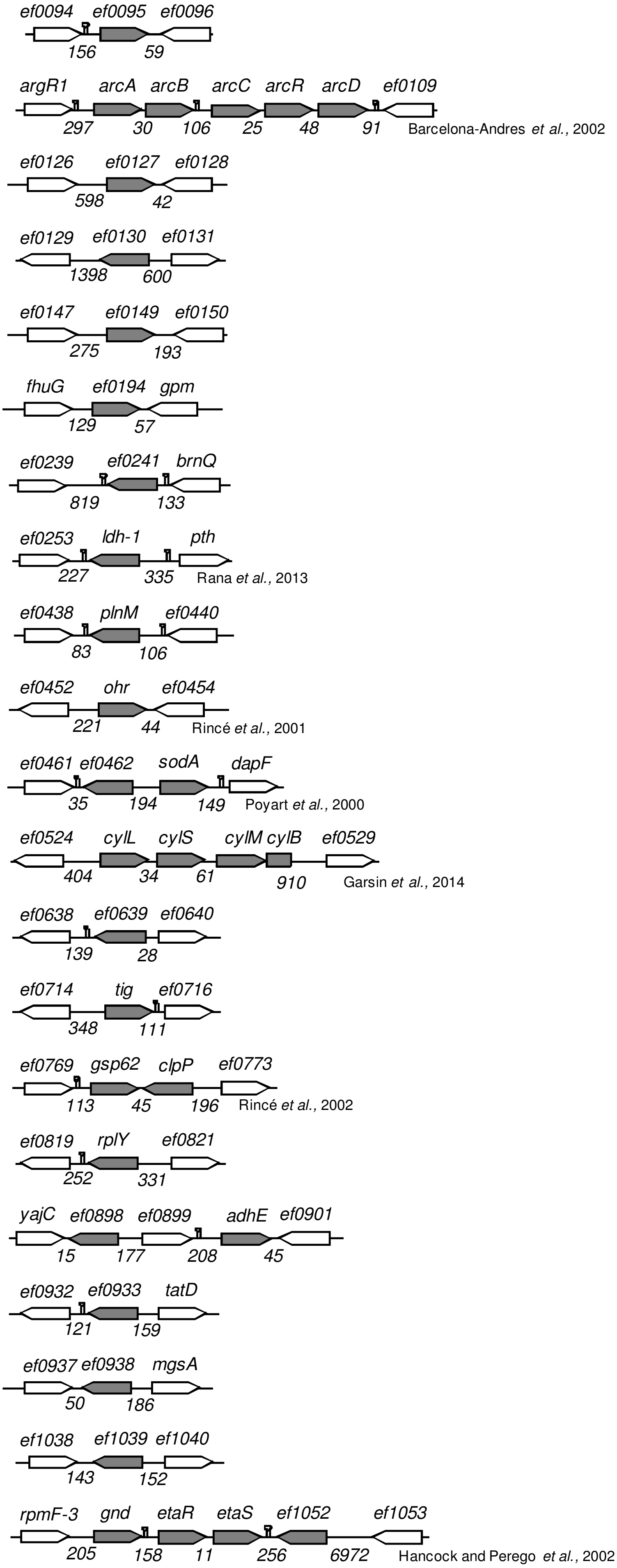
ef0094 ef0095 ef0096
156 59
argR1 arcA arcB arcC arcR arcD ef0109
297 30 106 25 48 91 Barcelona-Andres et al., 2002
ef0126 ef0127 ef0128
598 42
ef0129 ef0130 ef0131
1398 600
ef0147 ef0149 ef0150
275 193
fhuG ef0194 gpm
129 57
ef0239 ef0241 brnQ
819 133
ef0253 ldh-1 pth
227 335 Rana et al., 2013
ef0438 plnM ef0440
83 106
ef0452 ohr ef0454
221 44 Rincé et al., 2001
ef0461 ef0462 sodA dapF
35 194 149 Poyart et al., 2000
ef0524 cylL cylS cylM cylB ef0529
404 34 61 910 Garsin et al., 2014
ef0638 ef0639 ef0640
139 28
ef0714 tig ef0716
348 111
ef0769 gsp62 clpP ef0773
113 45 196 Rincé et al., 2002
ef0819 rplY ef0821
252 331
yajC ef0898 ef0899 adhE ef0901
15 177 208 45
ef0932 ef0933 tatD
121 159
ef0937 ef0938 mgsA
50 186
ef1038 ef1039 ef1040
143 152
rpmF-3 gnd etaR etaS ef1052 ef1053
205 158 11 256 6972 Hancock and Perego et al., 2002

Figure S1-A

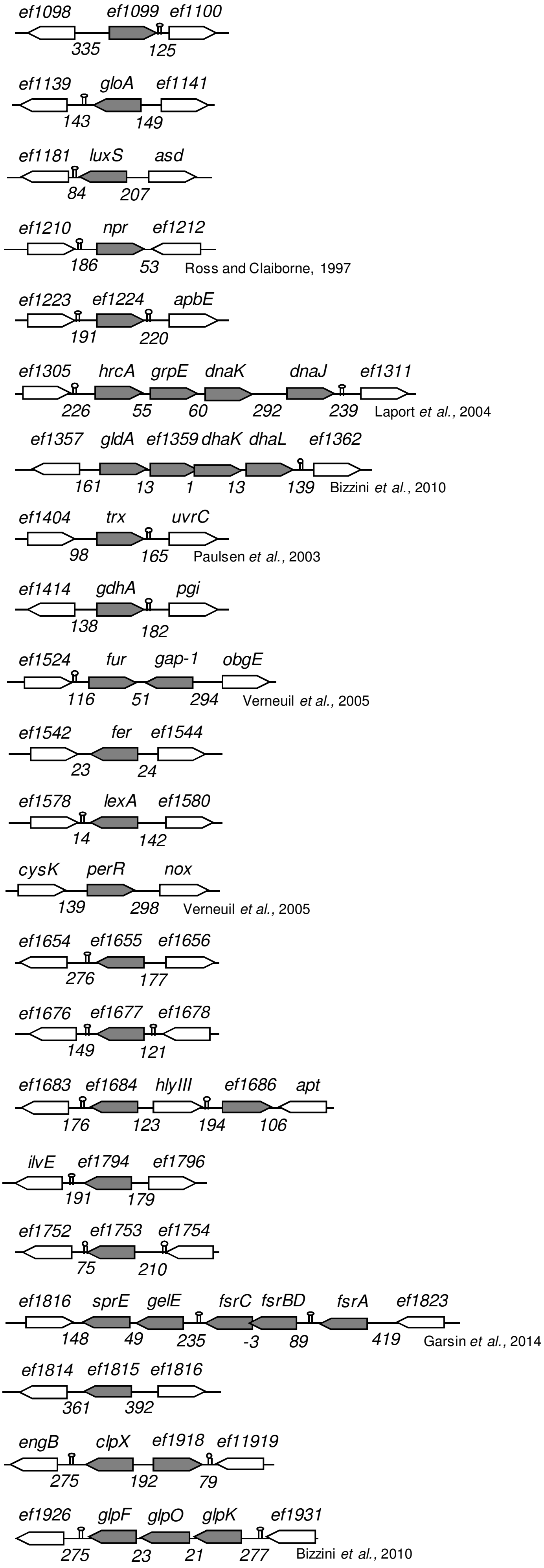
ef1098 ef1099 ef1100
335 125
ef1139 gloA ef1141
143 149
ef1181 luxS asd
84 207
ef1210 npr ef1212
186 53 Ross and Claiborne, 1997
ef1223 ef1224 apbE
191 220
ef1305 hrcA grpE dnaK dnaJ ef1311
226 55 60 292 239 Laport et al., 2004
ef1357 gldA ef1359 dhaK dhaL ef1362
161 13 1 13 139 Bizzini et al., 2010
ef1404 trx uvrC
98 165 Paulsen et al., 2003
ef1414 gdhA pgi
138 182
ef1524 fur gap-1 obgE
116 51 294 Verneuil et al., 2005
ef1542 fer ef1544
23 24
ef1578 lexA ef1580
14 142
cysK perR nox
139 298 Verneuil et al., 2005
ef1654 ef1655 ef1656
276 177
ef1676 ef1677 ef1678
149 121
ef1683 ef1684 hlyIII ef1686 apt
176 123 194 106
ilvE ef1794 ef1796
191 179
ef1752 ef1753 ef1754
75 210
ef1816 sprE gelE fsrC fsrBD fsrA ef1823
148 49 235 -3 89 419 Garsin et al., 2014
ef1814 ef1815 ef1816
361 392
engB clpX ef1918 ef11919
275 192 79
ef1926 glpF glpO glpK ef1931
275 23 21 277 Bizzini et al., 2010

Figure S1-A

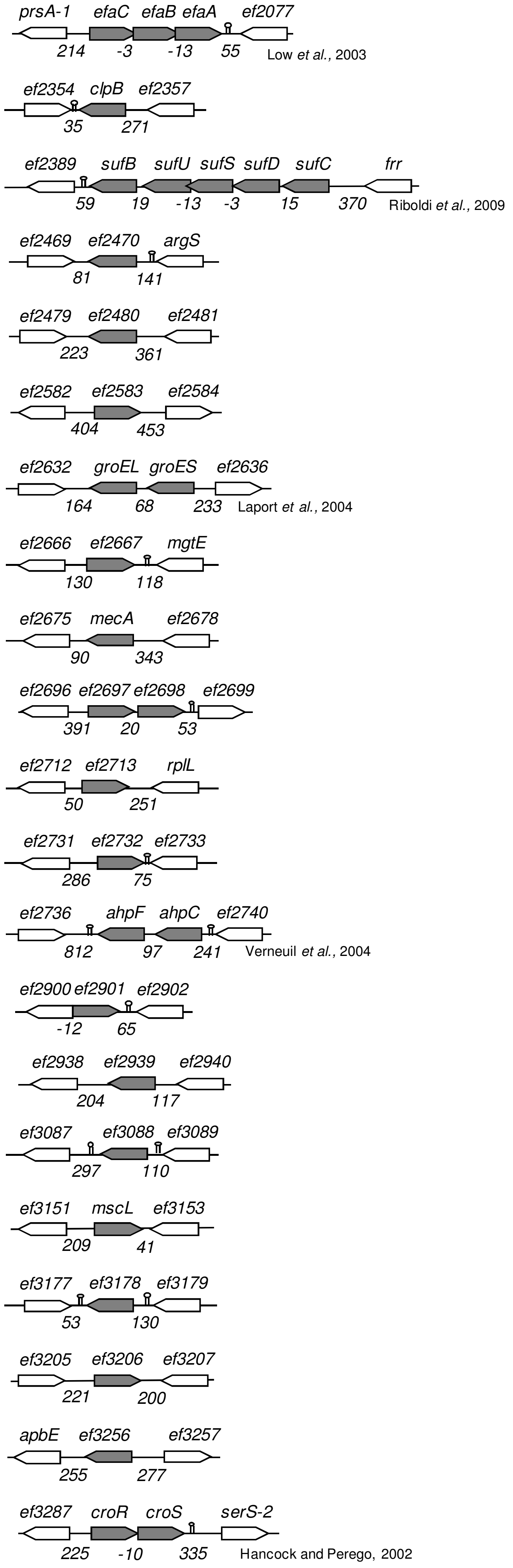
prsA-1 efaC efaB efaA ef2077
214 -3 -13 55
Low et al., 2003
ef2354 clpB ef2357
35 271
ef2389 sufB sufU sufS sufD sufC frr
59 19 -13 -3 15 370
Riboldi et al., 2009
ef2469 ef2470 argS
81 141
ef2479 ef2480 ef2481
223 361
ef2582 ef2583 ef2584
404 453
ef2632 groEL groES ef2636
164 68 233
Laport et al., 2004
ef2666 ef2667 mgtE
130 118
ef2675 mecA ef2678
90 343
ef2696 ef2697 ef2698 ef2699
391 20 53
ef2712 ef2713 rplL
50 251
ef2731 ef2732 ef2733
286 75
ef2736 ahpF ahpC ef2740
812 97 241
Verneuil et al., 2004
ef2900 ef2901 ef2902
-12 65
ef2938 ef2939 ef2940
204 117
ef3087 ef3088 ef3089
297 110
ef3151 mscL ef3153
209 41
ef3177 ef3178 ef3179
53 130
ef3205 ef3206 ef3207
221 200
apbE ef3256 ef3257
255 277
ef3287 croR croS serS-2
225 -10 335
Hancock and Perego, 2002

Figure S1-B

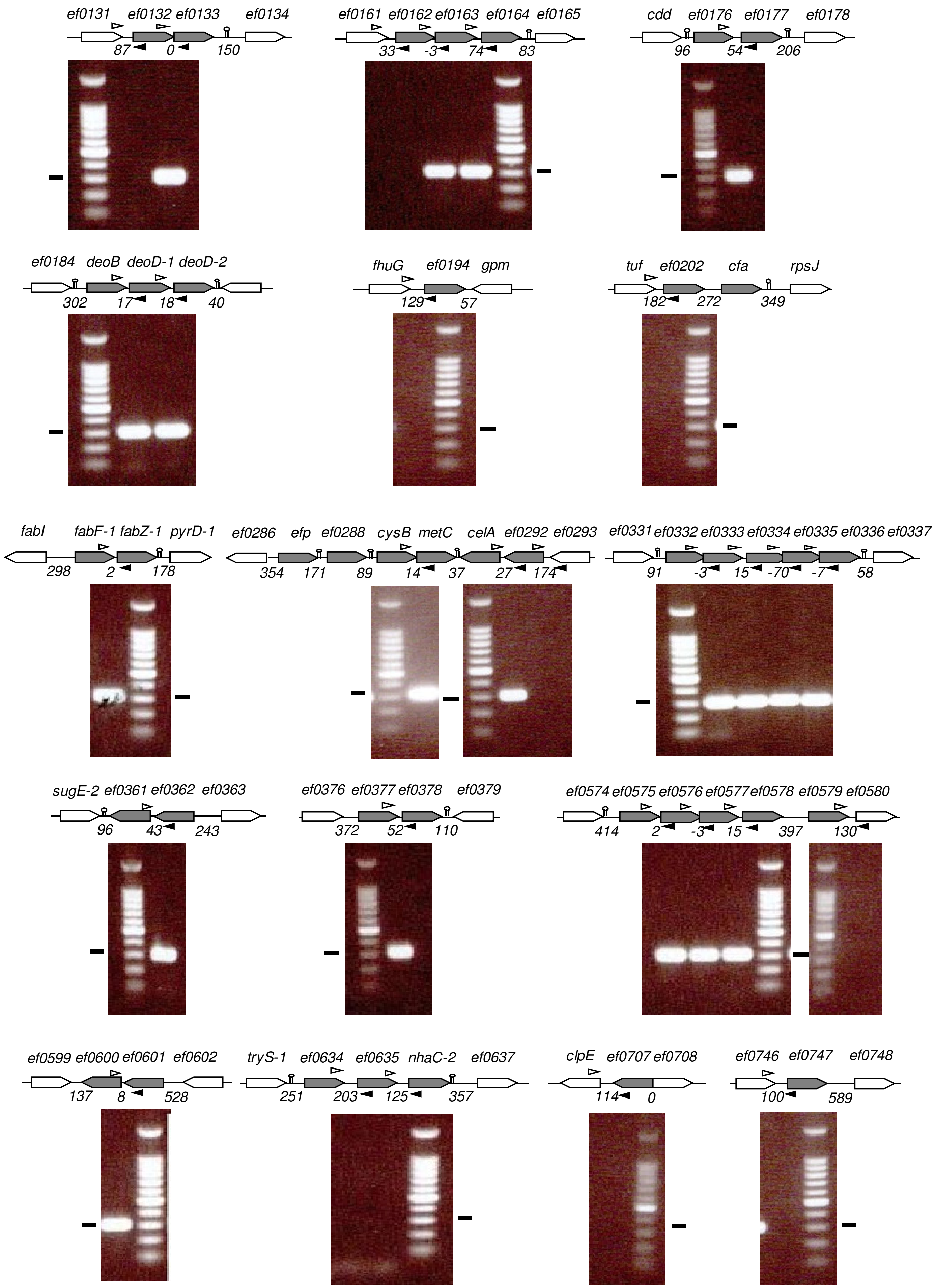
ef0131 ef0132 ef0133 ef0134
87 0 150
ef0161 ef0162 ef0163 ef0164 ef0165
33 -3 74 83
cdd ef0176 ef0177 ef0178
96 54 206
ef0184 deoB deoD-1 deoD-2
302 17 18 40
fhuG ef0194 gpm
129 57
tuf ef0202 cfa rpsJ
182 272 349
fabI fabF-1 fabZ-1 pyrD-1
298 2 178
ef0286 efp ef0288 cysB metC celA ef0292 ef0293
354 171 89 14 37 27 174
ef0331 ef0332 ef0333 ef0334 ef0335 ef0336 ef0337
91 -3 15 -70 -7 58
sugE-2 ef0361 ef0362 ef0363
96 43 243
ef0376 ef0377 ef0378 ef0379
372 52 110
ef0574 ef0575 ef0576 ef0577 ef0578 ef0579 ef0580
414 2 -3 15 397 130
ef0599 ef0600 ef0601 ef0602
137 8 528
tryS-1 ef0634 ef0635 nhaC-2 ef0637
251 203 125 357
clpE ef0707 ef0708
114 0
ef0746 ef0747 ef0748
100 589

Figure S1-B

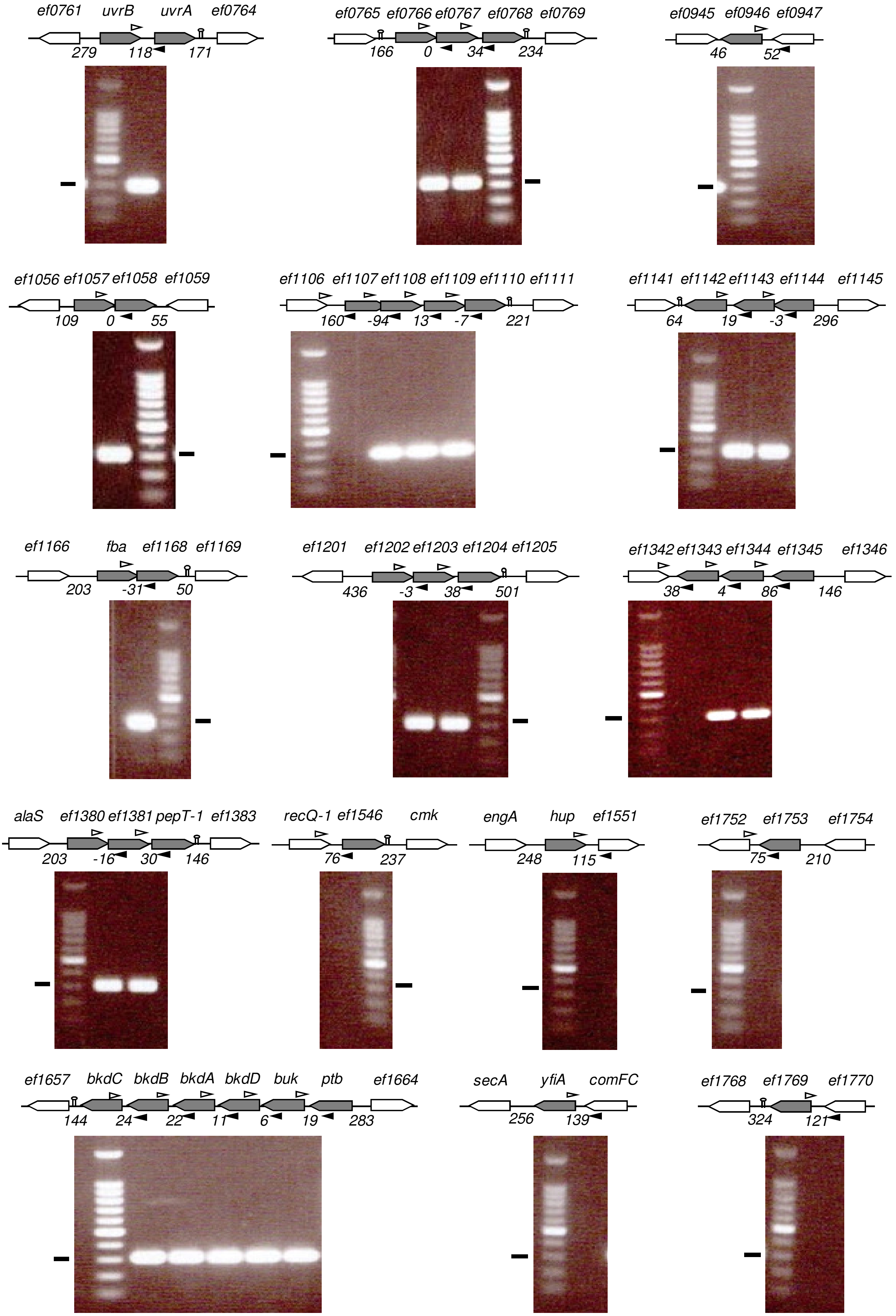
ef0761 uvrB uvrA ef0764
279 118 171
ef0765 ef0766 ef0767 ef0768 ef0769
166 0 34 234
ef0945 ef0946 ef0947
46 52
ef1056 ef1057 ef1058 ef1059
109 0 55
ef1106 ef1107 ef1108 ef1109 ef1110 ef1111
160 -94 13 -7 221
ef1141 ef1142 ef1143 ef1144 ef1145
64 19 -3 296
ef1166 fba ef1168 ef1169
203 -31 50
ef1201 ef1202 ef1203 ef1204 ef1205
436 -3 38 501
ef1342 ef1343 ef1344 ef1345 ef1346
38 4 86 146
alaS ef1380 ef1381 pepT-1 ef1383
203 -16 30 146
recQ-1 ef1546 cmk
76 237
engA hup ef1551
248 115
ef1752 ef1753 ef1754
75 210
ef1657 bkdC bkdB bkdA bkdD buk ptb ef1664
144 24 22 11 6 19 283
secA yfiA comFC
256 139
ef1768 ef1769 ef1770
324 121

Figure S1-B

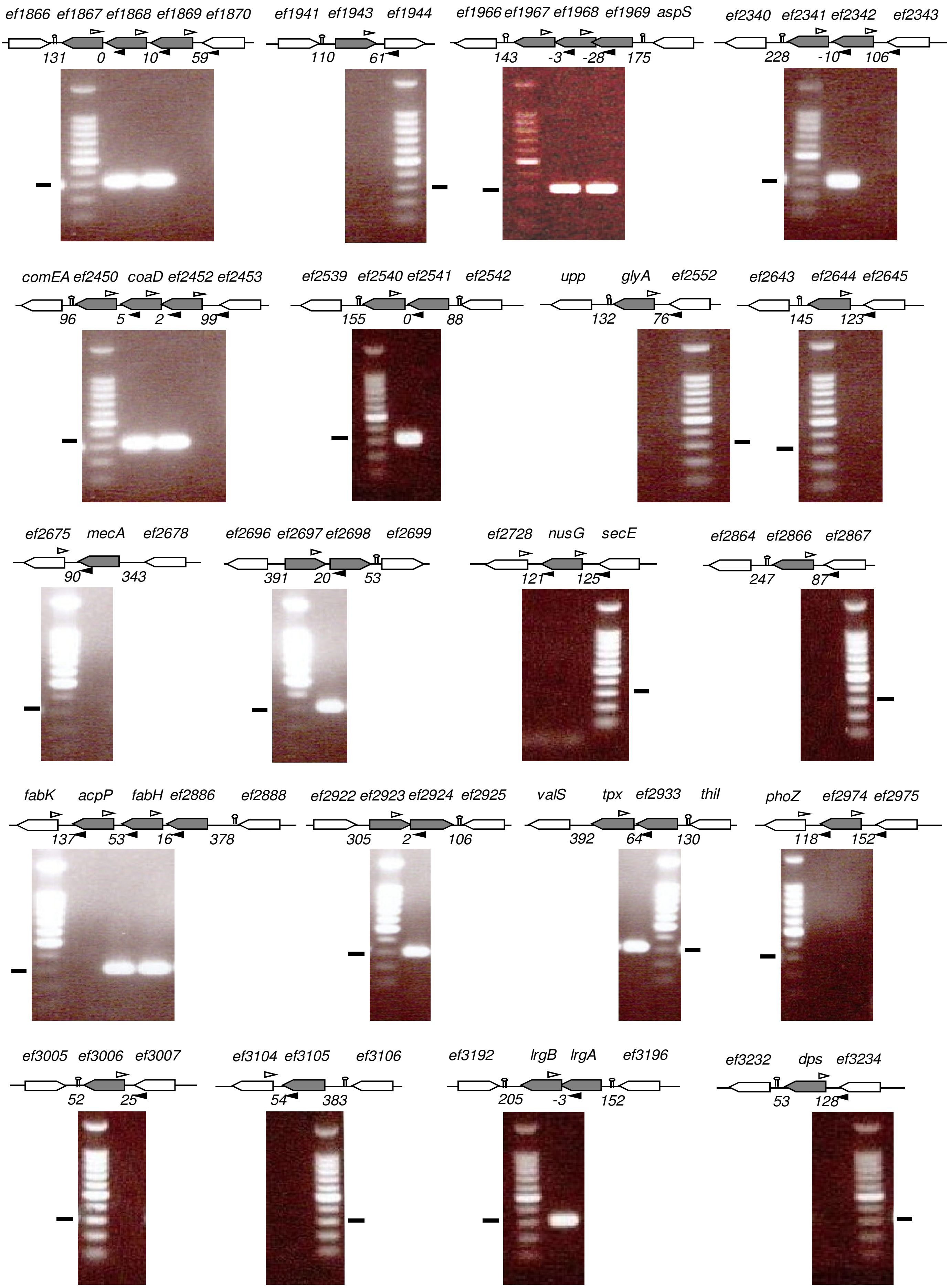
ef1866 ef1867 ef1868 ef1869 ef1870
131 0 10 59
ef1941 ef1943 ef1944
110 61
ef1966 ef1967 ef1968 ef1969 aspS
143 -3 -28 175
ef2340 ef2341 ef2342 ef2343
228 -10 106
comEA ef2450 coaD ef2452 ef2453
96 5 2 99
ef2539 ef2540 ef2541 ef2542
155 0 88
upp glyA ef2552
132 76
ef2643 ef2644 ef2645
145 123
ef2675 mecA ef2678
90 343
ef2696 ef2697 ef2698 ef2699
391 20 53
ef2728 nusG secE
121 125
ef2864 ef2866 ef2867
247 87
fabK acpP fabH ef2886 ef2888
137 53 16 378
ef2922 ef2923 ef2924 ef2925
305 2 106
valS tpx ef2933 thiI
392 64 130
phoZ ef2974 ef2975
118 152
ef3005 ef3006 ef3007
52 25
ef3104 ef3105 ef3106
54 383
ef3192 lrgB lrgA ef3196
205 -3 152
ef3232 dps ef3234
53 128

Figure S1 – C

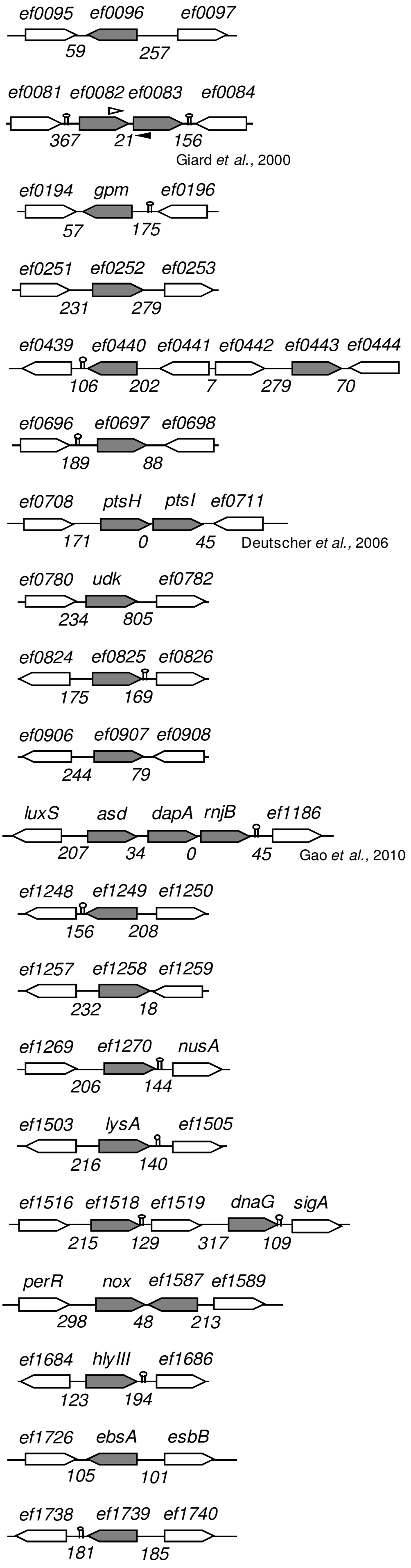
ef0095 ef0096 ef0097
59 257
ef0081 ef0082 ef0083 ef0084
367 21 156
Giard et al., 2000
ef0194 gpm ef0196
57 175
ef0251 ef0252 ef0253
231 279
ef0439 ef0440 ef0441 ef0442 ef0443 ef0444
106 202 7 279 70
ef0696 ef0697 ef0698
189 88
ef0708 ptsH ptsI ef0711
171 0 45
Deutscher et al., 2006
ef0780 udk ef0782
234 805
ef0824 ef0825 ef0826
175 169
ef0906 ef0907 ef0908
244 79
luxS asd dapA rnjB ef1186
207 34 0 45
Gao et al., 2010
ef1248 ef1249 ef1250
156 208
ef1257 ef1258 ef1259
232 18
ef1269 ef1270 nusA
206 144
ef1503 lysA ef1505
216 140
ef1516 ef1518 ef1519 dnaG sigA
215 129 317 109
perR nox ef1587 ef1589
298 48 213
ef1684 hlyIII ef1686
123 194
ef1726 ebsA esbB
105 101
ef1738 ef1739 ef1740
181 185

Figure S1-C

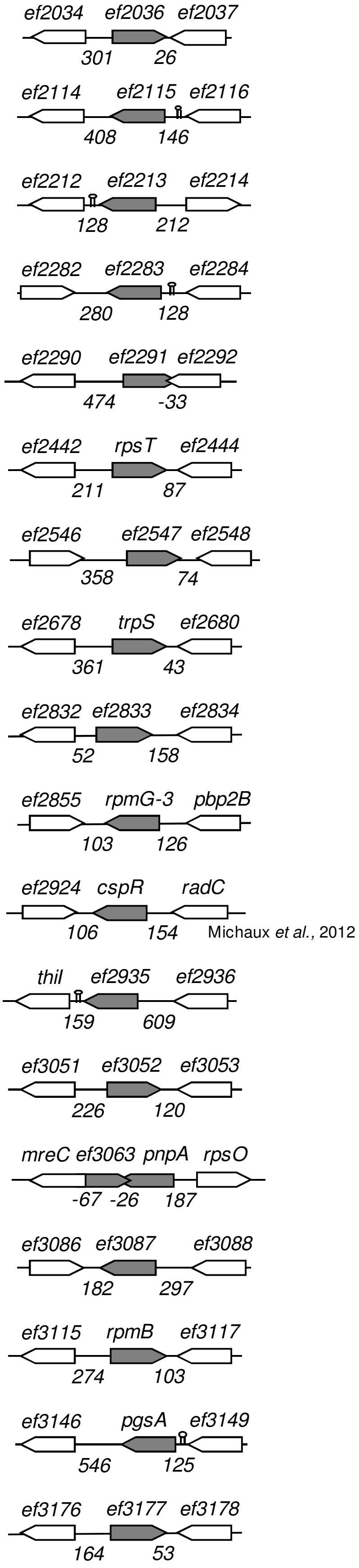

ef2034 ef2036 ef2037
301 26
ef2114 ef2115 ef2116
408 146
ef2212 ef2213 ef2214
128 212
ef2282 ef2283 ef2284
280 128
ef2290 ef2291 ef2292
474 -33
ef2442 rpsT ef2444
211 87
ef2546 ef2547 ef2548
358 74
ef2678 trpS ef2680
361 43
ef2832 ef2833 ef2834
52 158
ef2855 rpmG-3 pbp2B
103 126
ef2924 cspR radC
106 154
Michaux et al., 2012
thiI ef2935 ef2936
159 609
ef3051 ef3052 ef3053
226 120
mreC ef3063 pnpA rpsO
-67 -26 187
ef3086 ef3087 ef3088
182 297
ef3115 rpmB ef3117
274 103
ef3146 pgsA ef3149
546 125
ef3176 ef3177 ef3178
164 53

Figure S1-D

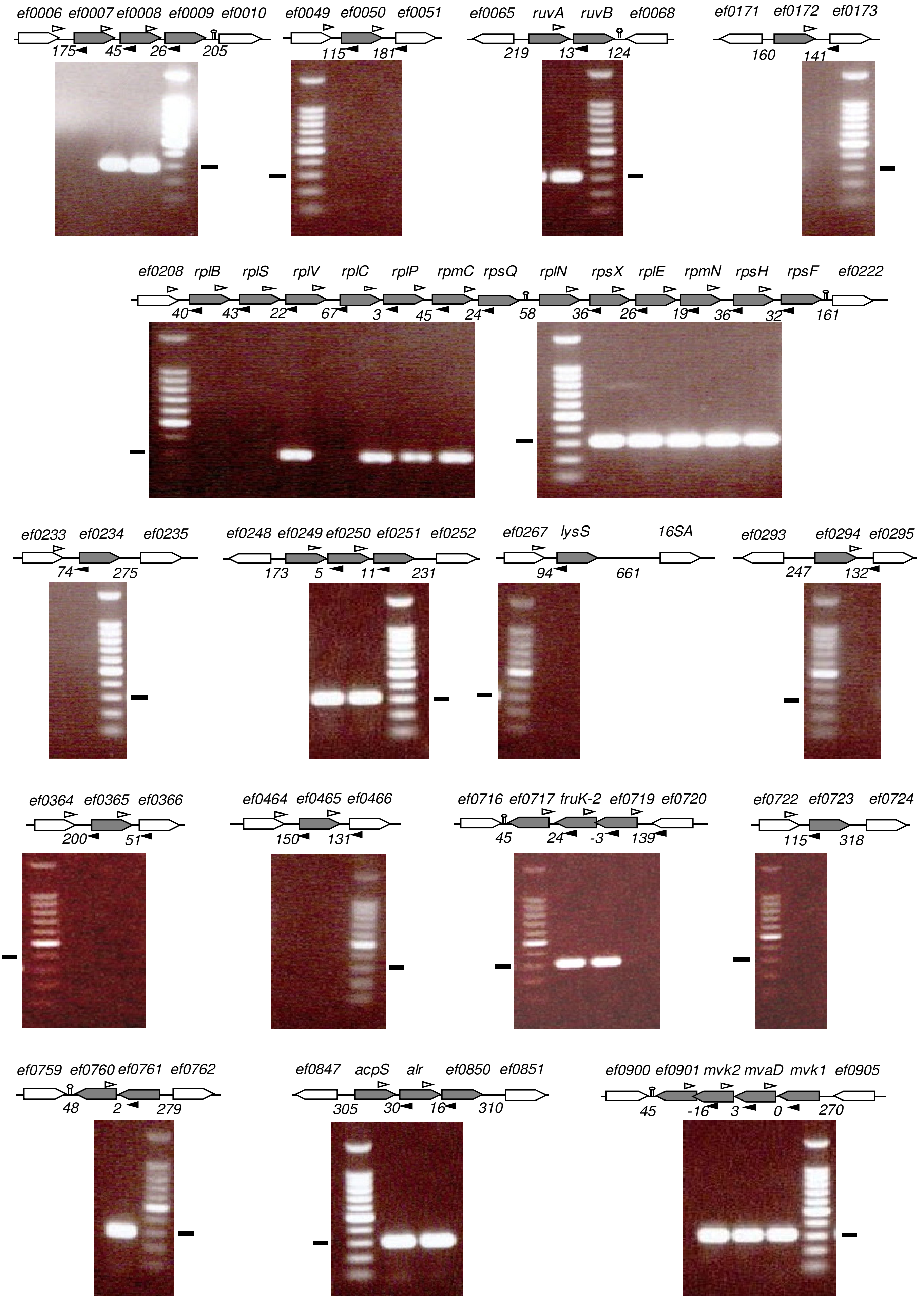
ef0006 ef0007 ef0008 ef0009 ef0010
175 45 26 205
ef0049 ef0050 ef0051
115 181
ef0065 ruvA ruvB ef0068
219 13 124
ef0171 ef0172 ef0173
160 141
ef0208 rplB rplS rplV rplC rplP rpmC rpsQ rplN rpsX rplE rpmN rpsH rpsF ef0222
40 43 22 67 3 45 24 58 36 26 19 36 32 161
ef0233 ef0234 ef0235
74 275
ef0248 ef0249 ef0250 ef0251 ef0252
173 5 11 231
ef0267 lysS 16SA
94 661
ef0293 ef0294 ef0295
247 132
ef0364 ef0365 ef0366
200 51
ef0464 ef0465 ef0466
150 131
ef0716 ef0717 fruK-2 ef0719 ef0720
45 24 -3 139
ef0722 ef0723 ef0724
115 318
ef0759 ef0760 ef0761 ef0762
48 2 279
ef0847 acpS alr ef0850 ef0851
305 30 16 310
ef0900 ef0901 mvk2 mvaD mvk1 ef0905
45 -16 3 0 270

Figure S1-D

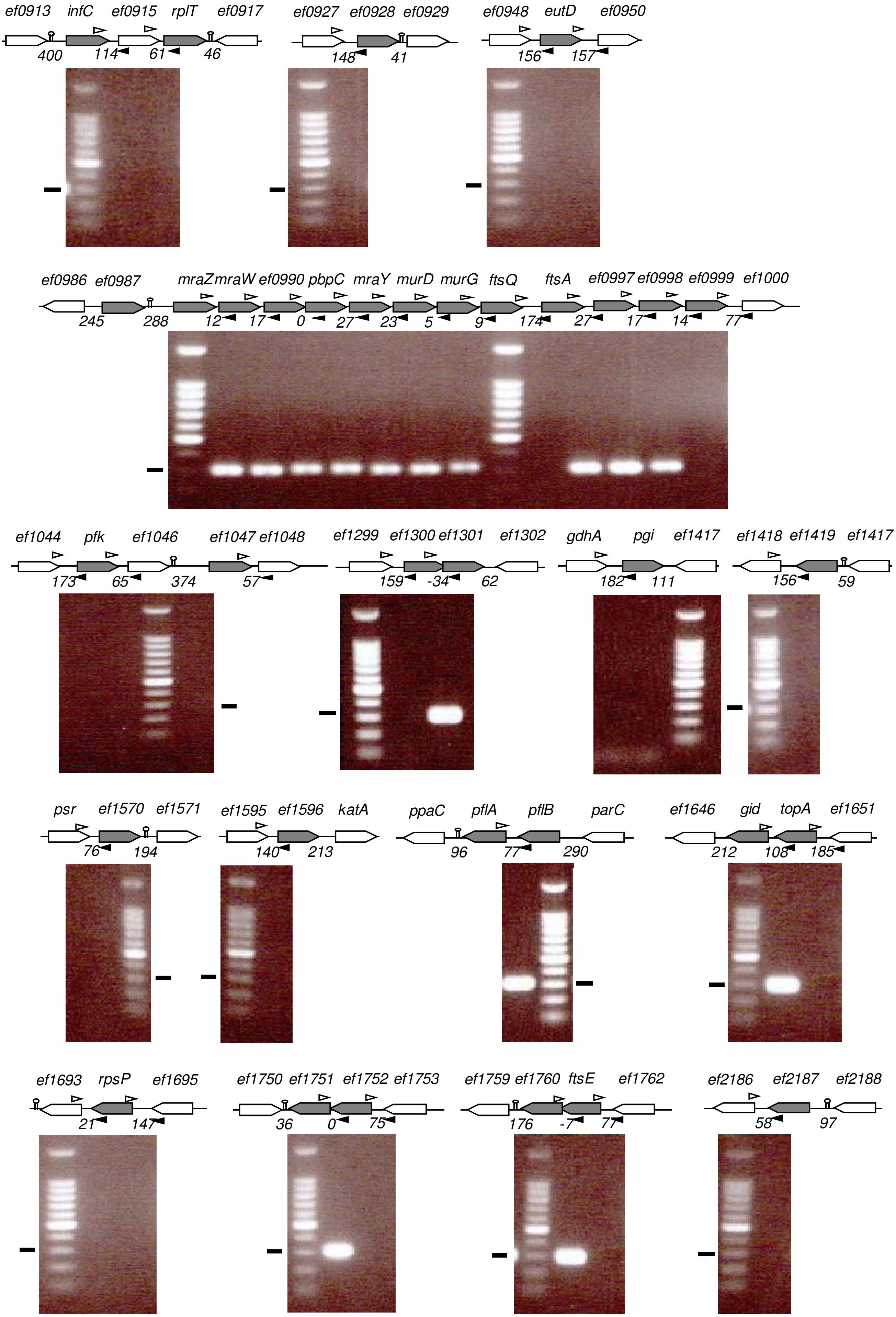
ef0913 infC ef0915 rplT ef0917
400 114 61 46
ef0927 ef0928 ef0929
148 41
ef0948 eutD ef0950
156 157
ef0986 ef0987 mraZ mraW ef0990 pbpC mraY murD murG ftsQ ftsA ef0997 ef0998 ef0999 ef1000
245 288 12 17 0 27 23 5 9 174 27 17 14 77
ef1044 pfk ef1046 ef1047 ef1048
173 65 374 57
ef1299 ef1300 ef1301 ef1302
159 -34 62
gdhA pgi ef1417
182 111
ef1418 ef1419 ef1417
156 59
psr ef1570 ef1571
76 194
ef1595 ef1596 katA
140 213
ppaC pflA pflB parC
96 77 290
ef1646 gid topA ef1651
212 108 185
ef1693 rpsP ef1695
21 147
ef1750 ef1751 ef1752 ef1753
36 0 75
ef1759 ef1760 ftsE ef1762
176 -7 77
ef2186 ef2187 ef2188
58 97

Figure S1-D

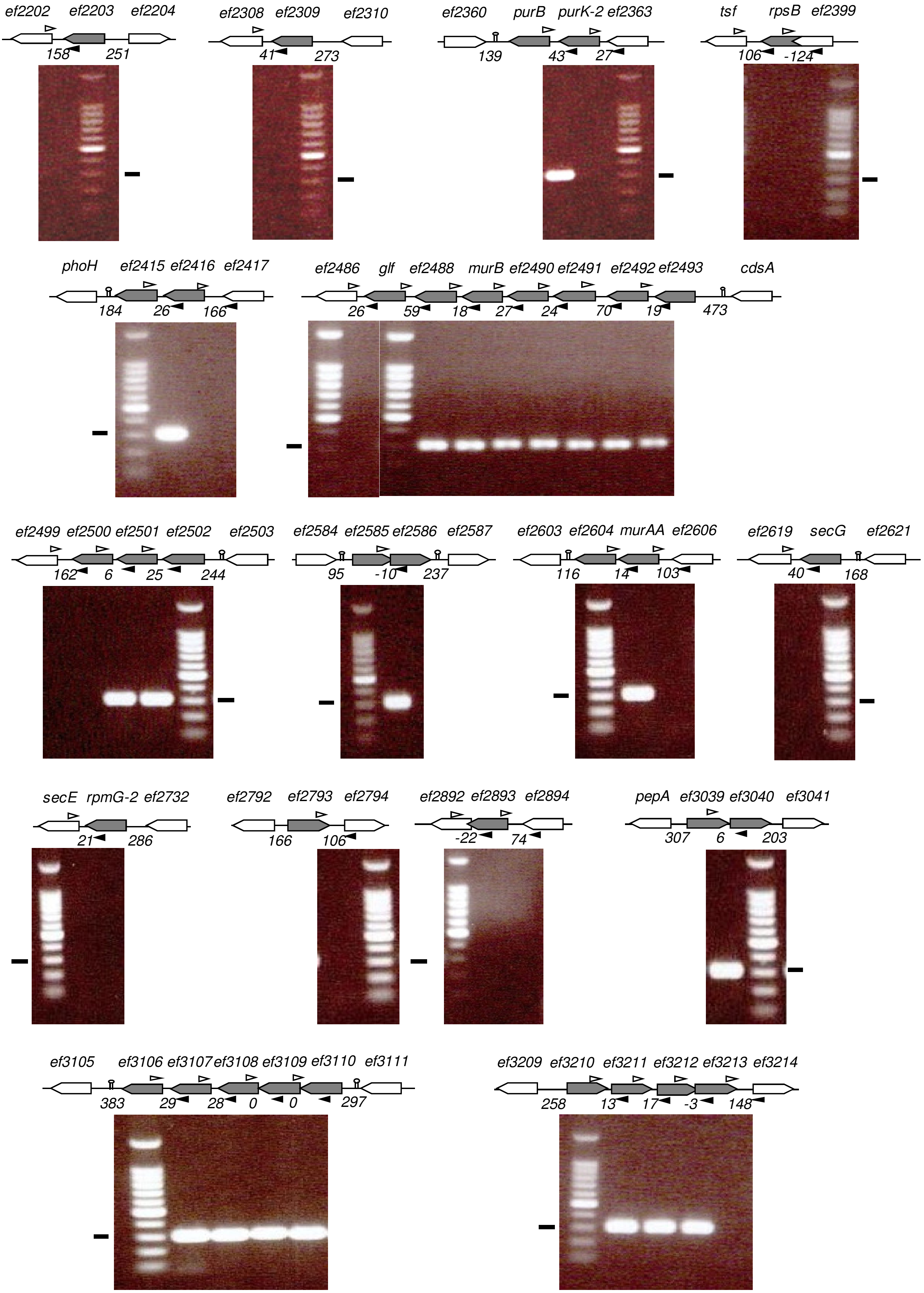

ef2202 ef2203 ef2204
158 251
ef2308 ef2309 ef2310
41 273
ef2360 purB purK-2 ef2363
139 43 27
tsf rpsB ef2399
106 -124
phoH ef2415 ef2416 ef2417
184 26 166
ef2486 glf ef2488 murB ef2490 ef2491 ef2492 ef2493 cdsA
26 59 18 27 24 70 19 473
ef2499 ef2500 ef2501 ef2502 ef2503
162 6 25 244
ef2584 ef2585 ef2586 ef2587
95 -10 237
ef2603 ef2604 murAA ef2606
116 14 103
ef2619 secG ef2621
40 168
secE rpmG-2 ef2732
21 286
ef2792 ef2793 ef2794
166 106
ef2892 ef2893 ef2894
-22 74
pepA ef3039 ef3040 ef3041
307 6 203
ef3105 ef3106 ef3107 ef3108 ef3109 ef3110 ef3111
383 29 28 0 0 297
ef3209 ef3210 ef3211 ef3212 ef3213 ef3214
258 13 17 -3 148

Supplemental references

Barcelona-Andrés B, Marina A, Rubio V (2002) Gene structure, organization, expression, and potential regulatory mechanisms of arginine catabolism in Enterococcus faecalis. J Bacteriol 184: 6289–6300.

Rana NF, Sauvageot N, Laplace J-M, Bao Y, Nes I, et al. (2013) Redox balance via lactate dehydrogenase is important for multiple stress resistance and virulence in Enterococcus faecalis. Infect Immun 81: 2662–2668. doi:10.1128/IAI.01299-12.

Rincé A, Giard JC, Pichereau V, Flahaut S, Auffray Y (2001) Identification and characterization of gsp65, an organic hydroperoxide resistance (ohr) gene encoding a general stress protein in Enterococcus faecalis. J Bacteriol 183: 1482–1488. doi:10.1128/JB.183.4.1482-1488.2001.

Poyart C, Quesnes G, Trieu-Cuot P (2000) Sequencing the gene encoding manganese-dependent superoxide dismutase for rapid species identification of enterococci. J Clin Microbiol 38: 415–418.

Garsin DA, Frank KL, Silanpää J, Ausubel FM, Hartke A, et al. (2014) Pathogenesis and Models of Enterococcal Infection. In: Gilmore MS, Clewell DB, Ike Y, Shankar N, editors. Enterococci: From Commensals to Leading Causes of Drug Resistant Infection. Boston: Massachusetts Eye and Ear Infirmary. Available: http://www.ncbi.nlm.nih.gov/books/NBK190426/. Accessed 25 June 2014.

Rincé A, Uguen M, Le Breton Y, Giard J-C, Flahaut S, et al. (2002) The Enterococcus faecalis gene encoding the novel general stress protein Gsp62. Microbiol Read Engl 148: 703–711

Hancock L, Perego M (2002) Two-Component Signal Transduction in Enterococcus faecalis. J Bacteriol 184: 5819–5825. doi:10.1128/JB.184.21.5819-5825.2002.

Ross RP, Claiborne A (1997) Evidence for regulation of the NADH peroxidase gene (npr) from Enterococcus faecalis by OxyR. FEMS Microbiol Lett 151: 177–183.

Laport MS, Lemos JAC, Bastos Md M do CF, Burne RA, Giambiagi-De Marval M (2004) Transcriptional analysis of the groE and dnaK heat-shock operons of Enterococcus faecalis. Res Microbiol 155: 252–258. doi:10.1016/j.resmic.2004.02.002.

Bizzini A, Zhao C, Budin-Verneuil A, Sauvageot N, Giard J-C, et al. (2010) Glycerol is metabolized in a complex and strain-dependent manner in Enterococcus faecalis. J Bacteriol 192: 779–785. doi:10.1128/JB.00959-09.

Paulsen IT, Banerjei L, Myers GSA, Nelson KE, Seshadri R, et al. (2003) Role of mobile DNA in the evolution of vancomycin-resistant Enterococcus faecalis. Science 299: 2071–2074. doi:10.1126/science.1080613.

Verneuil N, Rincé A, Sanguinetti M, Auffray Y, Hartke A, et al. (2005) Implication of hypR in the virulence and oxidative stress response of Enterococcus faecalis. FEMS Microbiol Lett 252: 137–141. doi:10.1016/j.femsle.2005.08.043.

Verneuil N, Rincé A, Sanguinetti M, Posteraro B, Fadda G, et al. (2005) Contribution of a PerR-like regulator to the oxidative-stress response and virulence of Enterococcus faecalis. Microbiol Read Engl 151: 3997–4004. doi:10.1099/mic.0.28325-0.

Low YL, Jakubovics NS, Flatman JC, Jenkinson HF, Smith AW (2003) Manganese-dependent regulation of the endocarditis-associated virulence factor EfaA of Enterococcus faecalis. J Med Microbiol 52: 113–119.

Riboldi GP, Verli H, Frazzon J (2009) Structural studies of the Enterococcus faecalis SufU [Fe-S] cluster protein. BMC Biochem 10: 3. doi:10.1186/1471-2091-10-3.

Verneuil N, Sanguinetti M, Le Breton Y, Posteraro B, Fadda G, et al. (2004) Effects of the Enterococcus faecalis hypR gene encoding a new transcriptional regulator on oxidative stress response and intracellular survival within macrophages. Infect Immun 72: 4424–4431. doi:10.1128/IAI.72.8.4424-4431.2004.

Giard JC, Rince A, Capiaux H, Auffray Y, Hartke A (2000) Inactivation of the stress- and starvation-inducible gls24 operon has a pleiotrophic effect on cell morphology, stress sensitivity, and gene expression in Enterococcus faecalis. J Bacteriol 182: 4512–4520.

Deutscher J, Francke C, Postma PW (2006) How phosphotransferase system-related protein phosphorylation regulates carbohydrate metabolism in bacteria. Microbiol Mol Biol Rev MMBR 70: 939–1031. doi:10.1128/MMBR.00024-06.

Gao P, Pinkston KL, Nallapareddy SR, van Hoof A, Murray BE, et al. (2010) Enterococcus faecalis rnjB is required for pilin gene expression and biofilm formation. J Bacteriol 192: 5489–5498. doi:10.1128/JB.00725-10.

Michaux C, Martini C, Shioya K, Ahmed Lecheheb S, Budin-Verneuil A, et al. (2012) CspR, a cold shock RNA-binding protein involved in the long-term survival and the virulence of Enterococcus faecalis. J Bacteriol 194: 6900–6908. doi:10.1128/JB.01673-12.
