# Supplementary figures and images for: The Intraperitoneal Transcriptome of the Opportunistic Pathogen Enterococcus faecalis in Mice
Source: PLoS One. 2015 May 15;10(5):e0126143. doi: 10.1371/journal.pone.0126143 (PMC4433114; doi:10.1371/journal.pone.0126143)

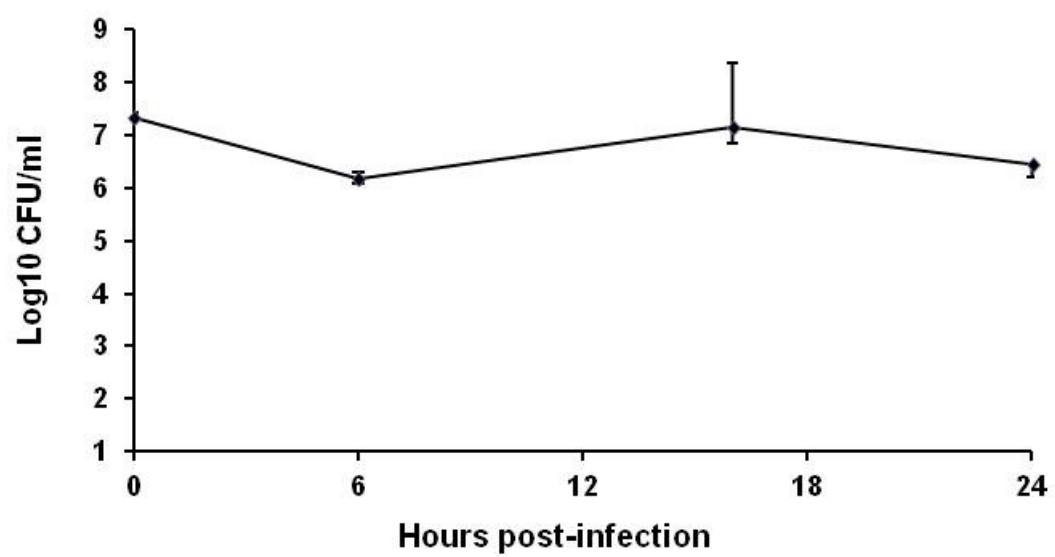

**Figure S2**

Supplement: S2 Fig — Mice were infected intraperitoneally with 2.108 CFU. The bacterial load was quantified by plating peritoneum wash sample dilutions in PBS onto BHI agar. The t0 time point represents the E. faecalis recovery 15 min after inoculation. The values represent the average of three biological replicates and error bars are indicated for each time point. (PDF) [file pone.0126143.s002.pdf]
